# Supplementary material for: Pexophagy suppresses ROS-induced damage in leaf cells under high-intensity light
Source: Nat Commun. 2022 Dec 5;13:7493. doi: 10.1038/s41467-022-35138-z (PMC9722907; doi:10.1038/s41467-022-35138-z)
Supplement: Supplementary file 1 — Supplementary Information file [file 41467_2022_35138_MOESM1_ESM.pdf]

Supplementary information

Pexophagy suppresses ROS-induced damage in leaf cells under high-intensity light

Kazusato Oikawa, Shino Goto-Yamada, Yasuko Hayashi, Daisuke Takahashi, Yoshitaka Kimori, Michitaro Shibata, Kohki Yoshimoto, Atsushi Takemiya, Maki Kondo, Kazumi Hikino, Akira Kato, Keisuke Shimoda, Haruko Ueda, Matsuo Uemura, Keiji Numata, Yoshinori Ohsumi, Ikuko Hara-Nishimura, Shoji Mano, Kenji Yamada and Mikio Nishimura

Corresponding authors

Kenji Yamada; kenji.yamada@uj.edu.pl

Mikio Nishimura; mramushini@gmail.com

Supplementary Methods

Transient expression analysis of ATG8

We transiently expressed the CFP-ATG8e construct in protoplast derived from 3-week-old *atg2(p1)* and *atg7* (*p4*) plant leaves expressing both RFP-PTS1 and ATG18a-GFP (Supplementary Figs. 6, 7). The protoplast transformation followed the previously described methods via PEG-mediated gene transfer[80,81]. The plasmid vector pUGW44-CFP-AtATG8e was constructed using the Gateway system (Thermo Fisher Scientific, Waltham, MA, USA) following **Vector construction** in **Methods.** Adapter-tagged cDNA of AtATG8e (At2g45170, accession no. NM_2055537) was generated using PCR by amplifying the corresponding region using the following primer set: F: 5'-AAAAAGCAGGCTTCATGAATAAAGGAAGCATCTT-3', R: 5'-AGAAAGCTGGGTTTAGA TTGAAGAAGCACCGA-3' for ATG8e, which was then recombined with the pDONR™221 vector[71]. Images were captured with a confocal laser scanning microscope (LSM 510 and LSM880, Zeiss, Germany) with a 40× or 63× objective. The excitation and emission wavelengths for the images were 405 nm and 450–470 nm, respectively, for CFP, 488 nm and 492–570 nm, respectively, for GFP, 516 nm and 600–625 nm, respectively, for RFP, and 488 nm and 670–730 nm, respectively, for chlorophyll autofluorescence. Co-localisation was determined from the merged image of the two or three fluorescence colours.

Ectopic expression of catalase

We transiently expressed *GFP-Cat2* or *RFP-Cat2* in the leaves of 3-week-old wild-type and *atg2(p1)* plants expressing RFP-PTS1 or GFP-PTS1, respectively (Supplementary Fig. 23). The plasmid vectors pGFPCAT2 or pRFPCAT2 were constructed using the Gateway-technology system (Thermo Fisher Scientific) following **Vector construction** in **Methods.** Adapter-tagged cDNA of *AtCat2* (At1g20620, accession no.NM_2034357) was generated using PCR by amplifying the corresponding region using the following primer set: CAT2-F: 5'-AAAAAGCAGGCTTTATGGAT CCTTACAAGTATCGTC-3' and CAT2-R: 5'-AGAAAGCTGGGTTTAGATGCTTGGTCTCAC GTT-3', which was then recombined with the pDONR™221 vector [71] generating pDONRCAT2. Images were captured with a confocal laser scanning microscope (LSM 510, Zeiss) with a 40× or 63× objective. The excitation and emission wavelengths for the images were 488 nm and 492–570 nm, respectively, for GFP, and 516 nm and 600–625 nm, respectively, for RFP. The number of peroxisomes in cells with or without expression of GFP-Cat2 or RFP-Cat2 was counted using cell counter plugins equipped in Image J Fiji after being adapted to high-intensity light (500 μmol m^{-2} s^{-1}) with an LED equipment (blue and red light) for 8 h. We used 500 μmol m^{-2} s^{-1} for preventing excess damage to the cutting leaves after particle bombardment.

ATG18 overexpression analysis

Plant growth test and H_2-DCF-DA staining of ATG18a-GFP-overexpressing lines were performed following the protocol provided in **Methods** (Supplementary Fig. 32)**.** We selected the two different ATG18a-GFP-overexpressing lines based on the fluorescence intensity of GFP. Three-week-old plants were stained with H_2-DCF after adaptation to high-intensity light (1,000 μmol m^{-2} s^{-1}) with an LED equipment (blue and red light) for 16 h.

NaCl treatment

One week after germination on 0.8% (w/v) agar plates containing half-strength MS medium and 1% (w/v) sucrose at 23 °C in a 16 h white light (100 μmol m^{-2} s^{-1})/8 h dark photoperiod, the wild type, *atg2*(*p1*), and *atg7*(*p4*) plants were transferred to the above medium containing 100 mM NaCl under the same light conditions (OSRAM FL25W White, Hitachi, Japan) at the same photoperiod for 2 weeks. The plant growth test and imaging analysis of peroxisomes and peroxisome aggregates (Supplementary Figs. 33, 34) were conducted following the **Methods**.

Wortmannin treatment

Rosette leaves of a 3-week-old *atg2*(*p1*) plant expressing ATG18a-GFP or GFP-2×FYVE and *atg7*(*p4*) plant expressing ATG18a-GFP were submerged in 20 mM wortmannin (Sigma-Aldrich, St. Louise, MO, USA) solution for 2 h under white light conditions (100 μmol m^{-2} s^{-1}), and subsequently they were observed with a confocal laser scanning microscope (LSM 510, Zeiss) (Supplementary Fig. 19). In the case of *atg7*(*p4*) plant expressing ATG18a-GFP, we further examined the 20 mM wortmannin solution for 4 h. The number of peroxisome aggregates with or without ATG18a-GFP and GFP-2×FYVE was counted using cell counter plugins equipped in Image J Fiji.

Concanamycin A treatment

Rosette leaves of a 3-week-old plant were submerged and deaerated in 20 mM ConA (Sigma-Aldrich) solution for 3 h under low (100 μmol m^{-2} s^{-1})- or high (1000 μmol m^{-2} s^{-1})-intensity light conditions, and subsequently observed using a confocal laser scanning microscope (LSM 510, Zeiss) (Supplementary Fig. 24). Z-series of cell images were obtained to evaluate whether peroxisomes are inside the vacuole. The number of peroxisomes in the vacuole was counted using cell counter plugins equipped in Image J Fiji.

Vacuole isolation

Vacuoles were isolated using protoplasts[82] derived from a 3-week-old wild-type and *atg7*(*p4*) plant expressing both RFP-PTS1 and Venus-VAM3 or ATG18a-GFP after the high-intensity (1000 μmol m^{-2} s^{-1}) light treatment for 5 h (Fig. 6 and Supplementary Figs. 27–29). To release vacuoles from the protoplasts, the protoplasts were washed with 0.6 M mannitol at 10,500 × *g* for 4 s and then transferred to the solution containing 0.6 M mannitol with 25mM EDTA. The released vacuoles were isolated in supernatants after centrifugation at 10,500 × *g* for 10 s and were immediately observed with a confocal laser scanning microscopy (LSM 880, Zeiss). Time-lapse images were captured every five seconds for more than 1 min.

References

80. Oikawa, K. et al. Mitochondrial movement during its association with chloroplasts in Arabidopsis thaliana. *Commun. Biol.* 4, 292 (2021).
81. Yoo, S.-D., Cho, Y.-H. & Sheen, J. Arabidopsis mesophyll protoplasts: a versatile cell system for transient gene expression analysis. *Nat. Protoc.* 2, 1565–1572 (2007).
82. Nishimura, M. & Beevers, H. Hydrolases in vacuoles from castor bean endosperm. *Plant Physiol.* 62, 44–48 (1978).

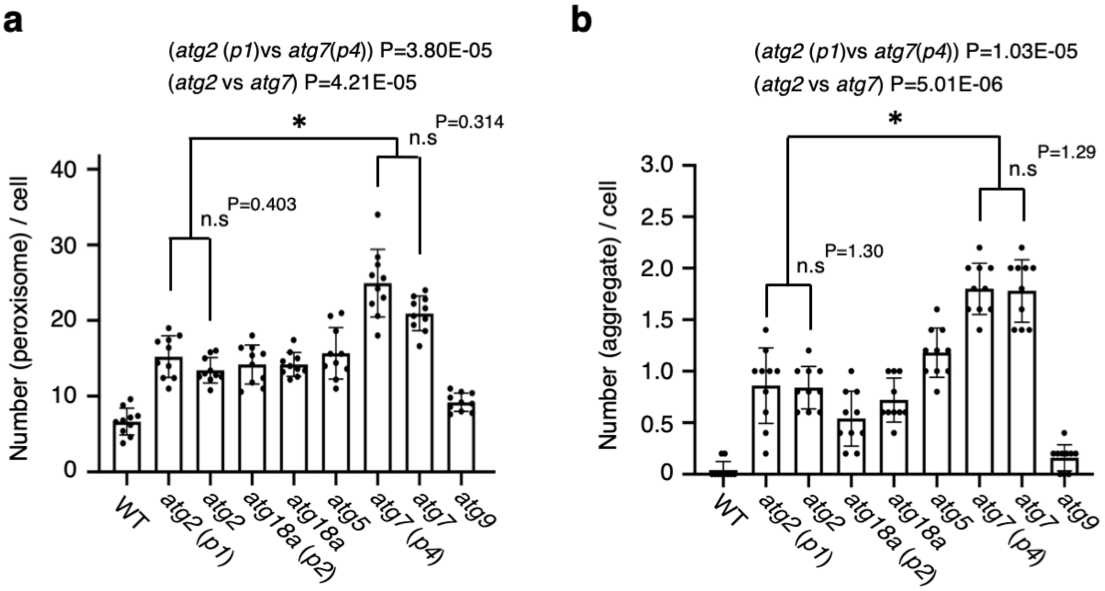

Supplementary Figure 1. The number of peroxisome and peroxisome aggregates in *atg* mutants

a, b, Number of peroxisomes (**a**) and peroxisome aggregates (**b**) per cell in leaf mesophyll cells from wild-type (WT) (GFP-PTS1), *atg2*/*peup1* (*atg2*(*p1*)), *atg2*, *atg18a*/*peup2* (*atg18a*(*p2*)), *atg18a, atg5, atg7/peup4* (*atg7*(*p4*)), *atg7, and atg9* plants. These plants were cultivated on an agar plate under normal white light (100 μmol m^{-2} s^{-1}). The images used for quantification were taken from the surface to middle region of a leaf mesophyll cell. More than 200 cells from ten images were examined. The error bars indicate mean ± standard deviation and asterisks indicate significant differences between *atg2* (*p1*) and *atg7*(*p4*), and between *atg2* and *atg7* in (**a**, **b**) under 100 μmol m^{-2} s^{-1} white light (**P* < 0.01, ***P* < 0.05, two-sided Student's *t*-test) (*n* = 10 biologically independent replicates**)**. There are no significant differences (n.s) between *atg2*(*p1*) and *atg2*, and between *atg7*(*p4*) and *atg7* in (**a**, **b**).

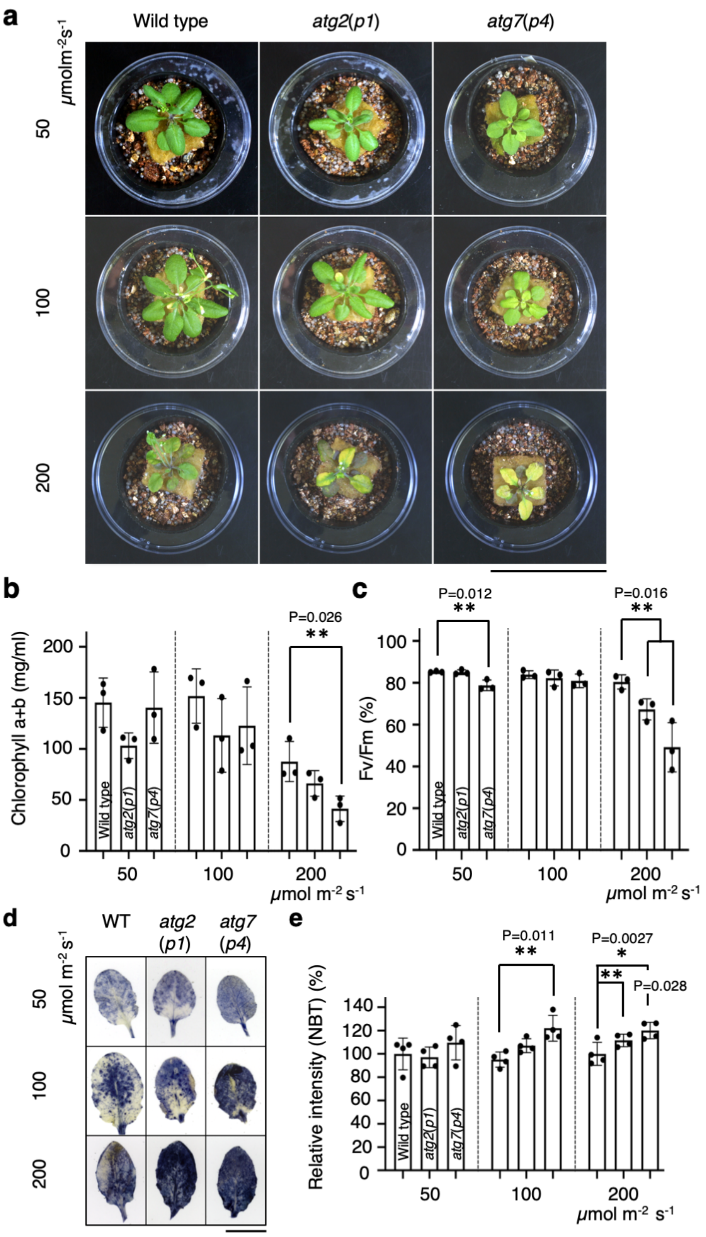

Supplementary Figure 2. Plant growth analysis of *atgs* under different light intensities

a, Growth of wild-type (WT), *atg2*(*p1*), and *atg7*(*p4*) plants under 50, 100, and 200 μmol m^{-2} s^{-1} light. Three-week-old plants cultured on rockwool inserted into the soil with ½ MS under low-intensity (50 μmol m^{-2} s^{-1}) white light conditions were used for the test ($n = 4$ technically independent replicates). Plants were then moved to each light condition for 2 days. Scale bar, 5 cm. **b**, **c,** Measurement of chlorophyll content (**b**) and photosynthetic efficiency (**c**) under the growth condition of (**a**). Three independent experiments were performed with three biological replicates. **d**, **e**, Nitroblue tetrazolium (NBT)-staining of representative leaf samples from WT, *atg2*(*p1*), and *atg7*(*p4*) plants (**d**) and relative quantification of NBT intensity using 15 leaves (**e**), in which WT in each light condition was set to 100%. Scale bar, 1 cm. The error bars indicate mean ± standard deviation ($n =$ 5 biologically independent replicates) and asterisks indicate significant differences between WT and *atg7*(*p4*) in (**b**), between WT and *atg2*(*p1*) or *atg7*(*p4*) in (**c**), between WT and *atg7*(*p4*) under 100 μmol m^{-2} s^{-1} white light in (**e**), and between WT and *atg2*(*p1*) or *atg7*(*p4*) under 200 μmol m^{-2} s^{-1} white light in (**e**) (*$P < 0.01$, **$P < 0.05$, two-sided Student's *t*-test).

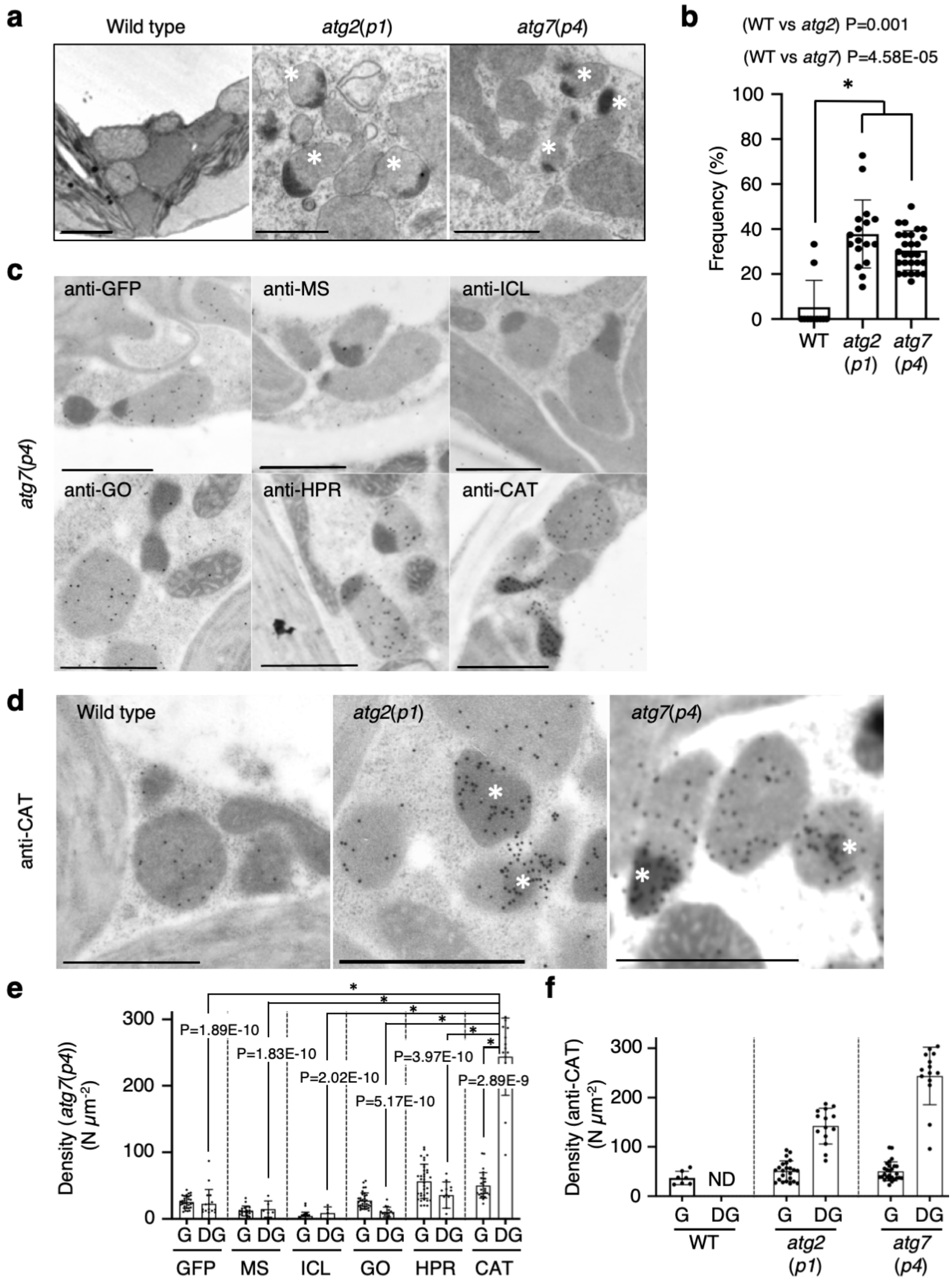

Supplementary Figure 3. Catalase accumulates in the high-density area of peroxisomes in *atg2* and *atg7*
a, Electron microscopy images of abnormal peroxisomes with high-density areas (dark grey, marked with white asterisks) in leaf mesophyll cells from *atg2*(*p1*) and *atg7*(*p4*) plants. These plants were cultivated on an agar plate under normal white light (100 µmol m^{-2} s^{-1}). Scale bars, 1 µm. **b**, Ratio of abnormal peroxisomes to total peroxisomes in the electron microscopic observation. Number of tested peroxisomes: wild type (WT) (n = 36), *atg2*(*p1*) (n = 134), and *atg7*(*p4*) (n = 183). The error bars indicate mean ± standard deviation (n = 5 technically independent sections) and asterisks indicate significant differences between WT and *atg2*(*p1*) or *atg7*(*p4*) (*P < 0.01, two-sided Student's t-test). **c**, Immunoelectron microscopy analyses performed using antibodies against GFP, malate synthase (MS), isocitrate lyase (ICL), glycolate oxidase (GO), hydroxypyruvate reductase (HPR), and catalase (CAT) in GFP-PTS1 expressing plants. Scale bars, 1 µm. **d**,

Enlarged images show that anti-CAT gold particles are highly accumulated in high-density areas (dark grey) of peroxisomes in *atg2*(*p1*) and *atg7*(*p4*) cells (white asterisks), but not in WT cells. Scale bars, 1 μm. **e**, Density of antibody-conjugated gold particles detected in grey (G) or dark-grey (DG) peroxisomes in (**c**). Number of tested peroxisomes: GFP (n = 32), MS (n = 22), ICL (n = 15), GO (n = 37), HPR (n = 35), and CAT (n = 30). The error bars indicate mean ± standard deviation and asterisks indicate significant differences between the density of each antibody detected in DG in *atg7*(*p4*) (*P < 0.01, two-sided Student's t-test). **f**, Density of antibody against CAT gold particles in G or DG peroxisomes from GFP-PTS1, *atg2*(*p1*), and *atg7*(*p4*) cells. Number of tested DG: GFP-PTS1 (n = 7), *atg2*(*p1*) (n = 14), and *atg7*(*p4*) (n = 23). The error bars indicate mean ± standard deviation (n = 5 technically independent sections). The electron microscopy images in (**a**, **c**–**d**) show a summary of the five independent sections.

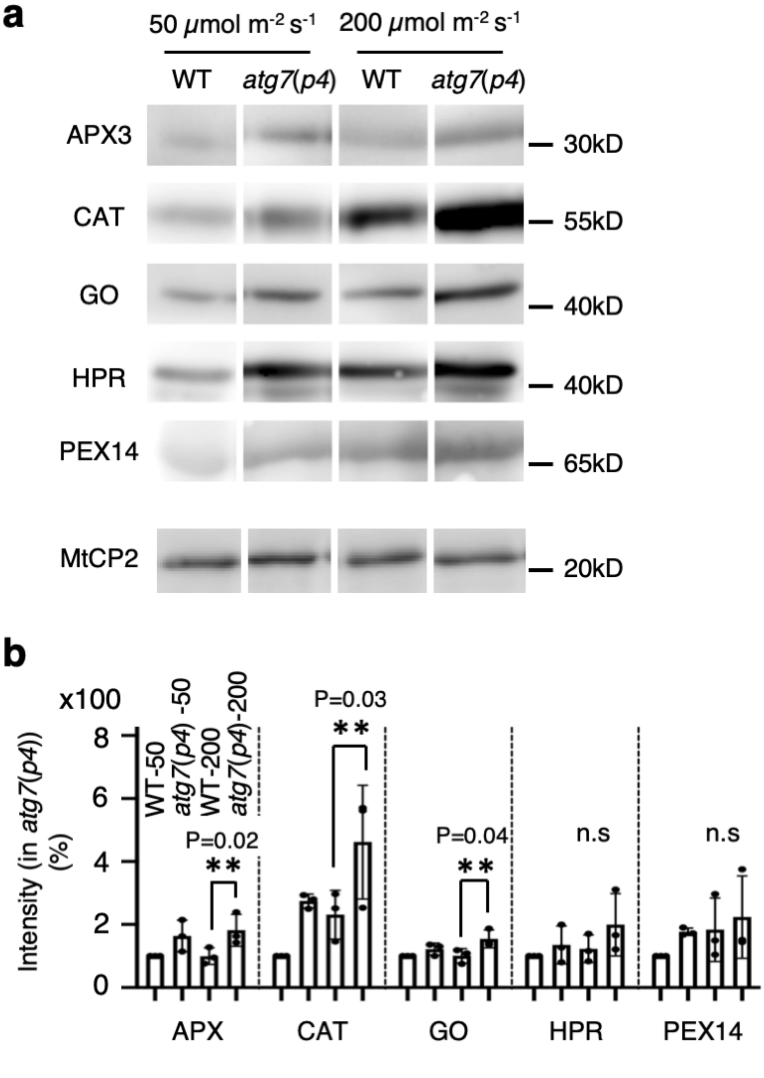

Supplementary Figure 4. High accumulation of catalase in *atg7* under light conditions

a, Immunoblotting analysis of the total protein extracted from the 50 or 200 µmol m^{-2} s^{-1} white light-adapted wild-type and *atg7*(*p4*) cells using antibodies against ascorbate peroxidase (APX3),catalase (CAT), glycolate oxidase (GO), hydroxypyruvate reductase (HPR), and peroxin 14 (PEX14) The representative images show a summary of at least three independent experiments. Mitochondrial tricyclic pyrone (MtCP2) was used as an internal control. These plants were cultivated on an agar plate under normal white light conditions (100 µmol m^{-2} s^{-1}) for two weeks and moved to each light condition for 3 d. **b,** Signal intensity of the immunoblotting analysis in (**a**)**.** The error bars indicate mean ± standard deviation and asterisks indicate significant differences between WT and *atg7*(*p4*) (n =3 technically independent experiments) (*$P < 0.01$, **$P < 0.05$, and n.s indicates not significant differences ($P > 0.05$), two-sided Student's *t*-test).

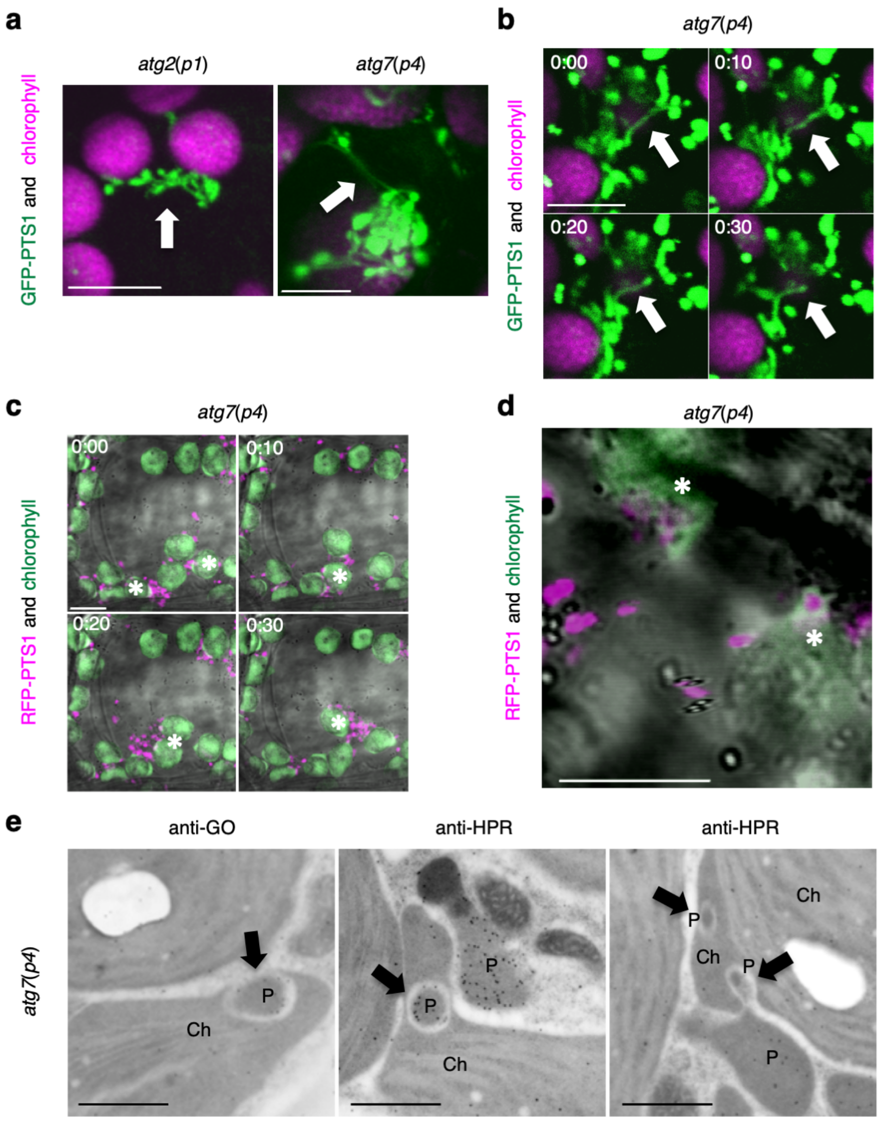

Supplementary Figure 5. Peroxules and stromules are generated in *atg7*

a, Peroxisomes (GFP-PTS1, green) and chloroplasts (chlorophyll autofluorescence, magenta) in leaf mesophyll cells from 100 µmol m^{-2} s^{-1} white light-adapted *atg2*(*p1*) and *atg7*(*p4*) plants. These plants were cultivated on an agar plate under the similar condition as in Figs. 1-3. **b**, Time-lapse images of peroxules extended from peroxisome aggregation in *atg7*(*p4*) obtained every 10 s. White arrows indicate peroxules in (**a**) and (**b**). **c**, Time-lapse images of engulfment (white asterisks) of peroxisomes (RFP-PTS1, magenta) by chloroplast membranes (autofluorescence, green) in *atg7*(*p4*) cells obtained every 10 s. **d**, An enlarged image shows peroxisomes in chloroplast membranes (white asterisks). Scale bars in (**a**–**d**), 10 µm. The representative images show a summary of at least eight independent experiments. **e**, Electron microscopy images of peroxisomes and chloroplasts in *atg7*(*p4*) cells. Arrows (black) indicate the engulfment of peroxisomes (P) by chloroplasts (Ch). Peroxisomes are identified using anti-glycolate oxidase (GO) or anti-hydroxypyruvate reductase (HPR) antibodies. Scale bars, 1 µm. The representative images show a summary of at least five independent experiments.

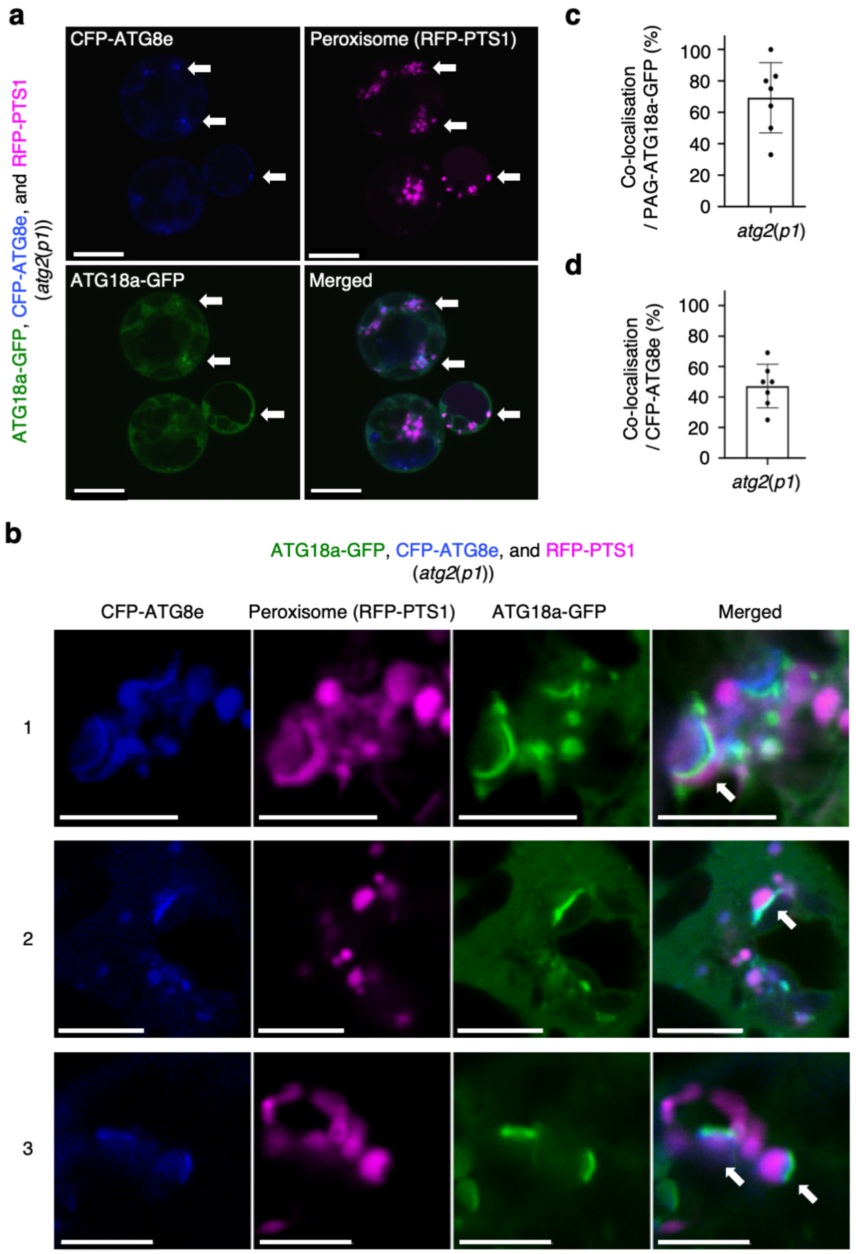

Supplementary Figure 6. ATG8e gathers on the peroxisome aggregates in *atg2* targeted by ATG18a-GFP

a, b, CLSM images of CFP-ATG8e (blue), peroxisomes (magenta), ATG18a-GFP (green), and a merged image of the three channels in protoplasts of *atg2*(*p1*) cells (**a**) and enlarged images (**b**). Scale bars are 10 µm in (**a**) and 5 µm in (**b**). White arrows indicate the co-localisation of ATG18a-GFP and CFP-ATG8e on peroxisomes in the peroxisome aggregate (PAG). CFP-ATG8e is transiently expressed in the protoplasts derived from leaf mesophyll cells of *atg2*(*p1*), which stably expresses RFP-PTS1 and ATG18a-GFP. Three-week-old plants cultured on an agar plate containing ½ MS with 1% sucrose under normal-intensity (100 µmol m^{-2} s^{-1}) white-light conditions were used. **c, d,** Ratio of the number of CFP-ATG8e and ATG18a-GFP co-localisation on PAG to the number of ATG18a-GFP on PAG (**c**) or the number of CFP-ATG8e targeted structures (**d**). The error bars indicate mean ± standard deviation (n = 7 biologically independent protoplasts). The representative images in (**a**, **b**) show a summary of at least 3 independent experiments.

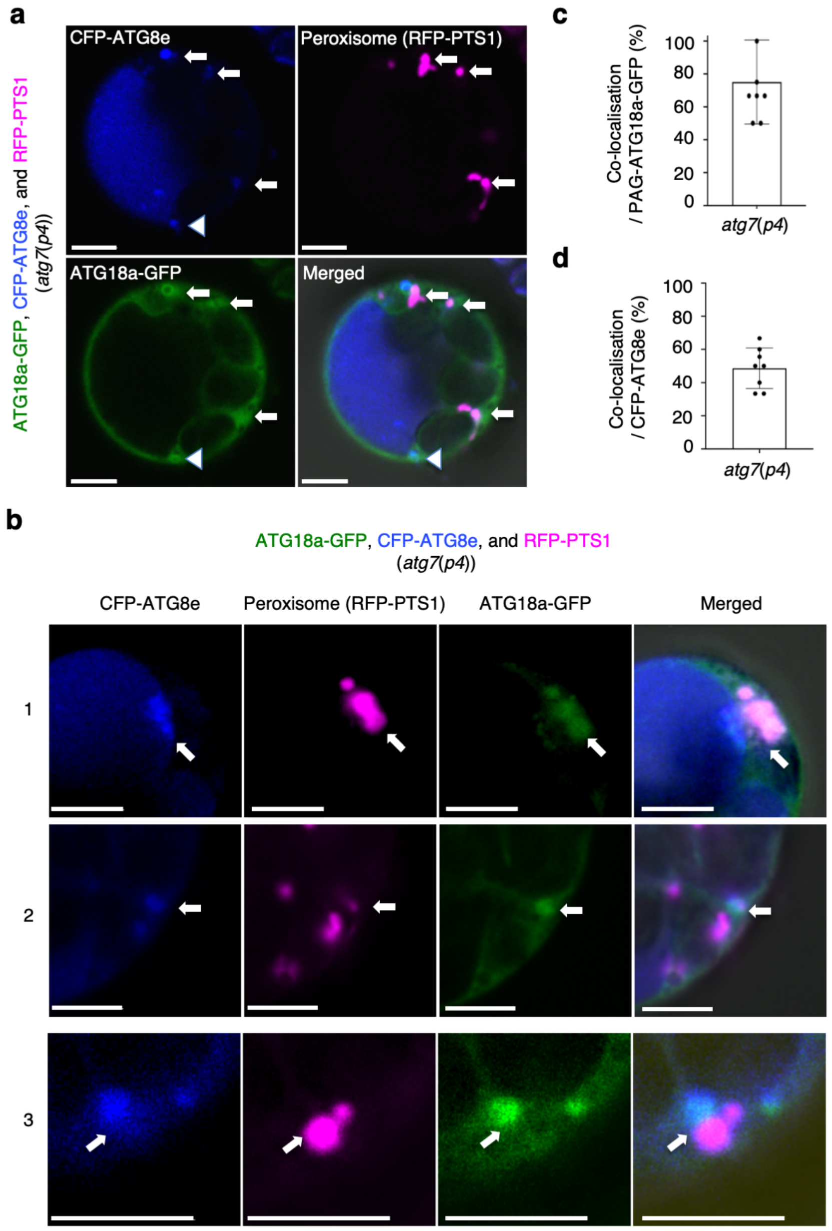

Supplementary Figure 7. ATG8e gathers on the peroxisome aggregates in *atg7* targeted by ATG18a-GFP

a, b, CLSM images of CFP-ATG8e (blue), peroxisomes (magenta), ATG18a-GFP (green), and a merged image of the three channels in protoplasts of *atg7*(*p4*) cells (**a**) and enlarged images (**b**). White arrows indicate the co-localisation of ATG18a-GFP and CFP-ATG8e on peroxisomes in peroxisome aggregates, and white-arrow heads indicate the co-localisation of ATG18a-GFP and CFP-ATG8e on an undefined structure. CFP-ATG8e is transiently expressed in the protoplasts derived from leaf mesophyll cells of *atg7*(*p4*), which stably expresses RFP-PTS1 and ATG18a-GFP. Three-week-old plants cultured on an agar plate containing ½ MS with 1% sucrose under normal-intensity (100 µmol m^{-2} s^{-1}) white-light conditions were used. Scale bars are 10 µm in (**a**) and 5 µm in (**b**). **c, d,** Ratio of the number of the CFP-ATG8e with ATG18a-GFP on PAG to the number of ATG18a-GFP on PAG (**c**) or the number of CFP-ATG8e targeted structure (**d**). The error bars indicate mean ± standard deviation (n = 7 biologically independent protoplasts). The representative images in (**a**, **b**) show a summary of four independent experiments.

a

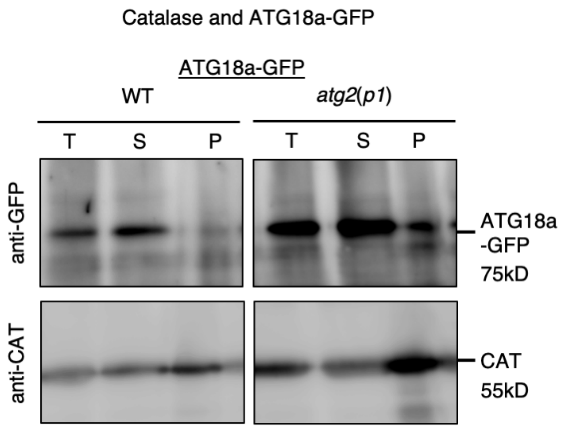

b

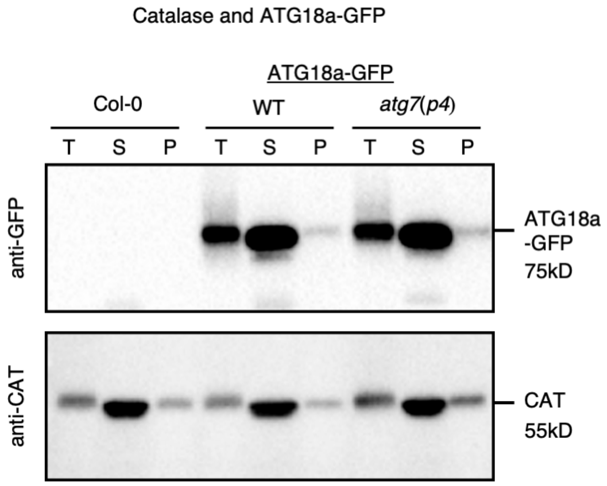

Supplementary Figure 8. Co-localisation of ATG18a-GFP and peroxisome in the pellet fraction

a, **b,** Immunoblotting analysis of catalase (CAT) and ATG18a-GFP in each fraction (total: T, supernatant: S, and pellet: P) of wild type (WT) expressing RFP-PTS1 and *atg2*(*p1*) expressing both RFP-PTS1 and ATG18a-GFP (**a**), Col-0, WT, and *atg7*(*p4*) expressing both RFP-PTS1 and ATG18a-GFP (**b**). The total protein was extracted from the three-week-old plants cultured on an agar plate containing ½ MS with 1% sucrose under normal-intensity (100 μmol m^{-2} s^{-1}) white-light conditions. The representative images in (**a**, **b**) show a summary of three independent experiments.

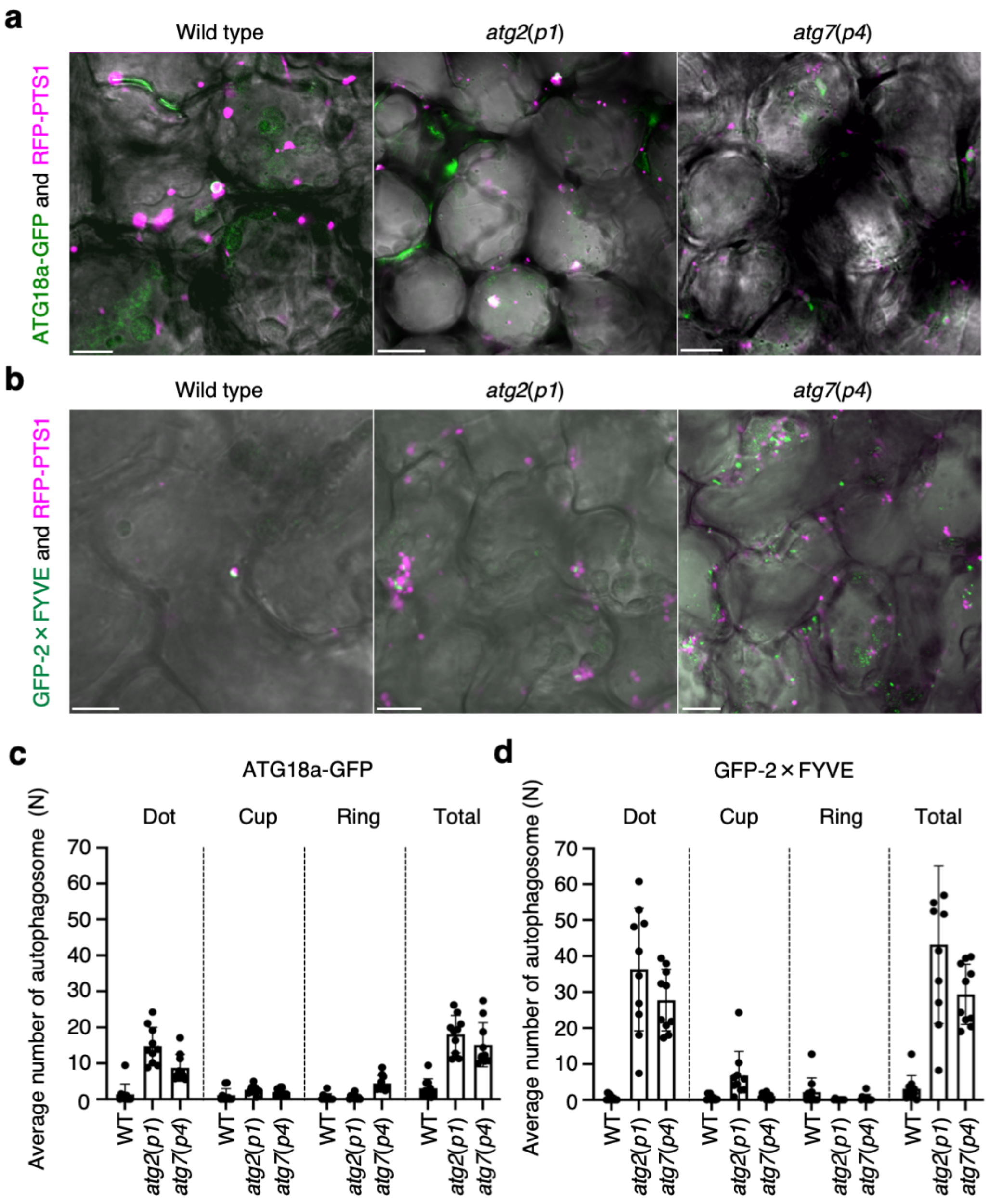

Supplementary Figure 9. Quantification of ATG18a-GFP and GFP-2×FYVE targeting peroxisomes in *atg2* and *atg7*

a, **b**, Representative images used for statistical analysis of peroxisomes in leaf mesophyll cells from wild-type, *atg2*(*p1*), and *atg7*(*p4*) plants targeted by ATG18a-GFP (**a**) or GFP-2×FYVE (**b**). These plants were cultured in the same condition as Figs. 1-3. Scale bars, 10 µm. **c**, **d**, The number of ATG18a-GFP or GFP-2×FYVE structures on peroxisomes. Structures are categorised into three types: dot, cup, and ring. The data are taken from ten sections and shown as the density at 100 × 100 μm^2. The number of three patterns for ATG18a-GFP (**c**) or GFP-2×FYVE (**d**) (**Supplementary Table 1**) are shown as a graph. The error bars indicate mean ± standard deviation (n = 10 biologically independent images) in (**c**, **d**). The representative images in (**a**, **b**) show a summary of eight independent experiments.

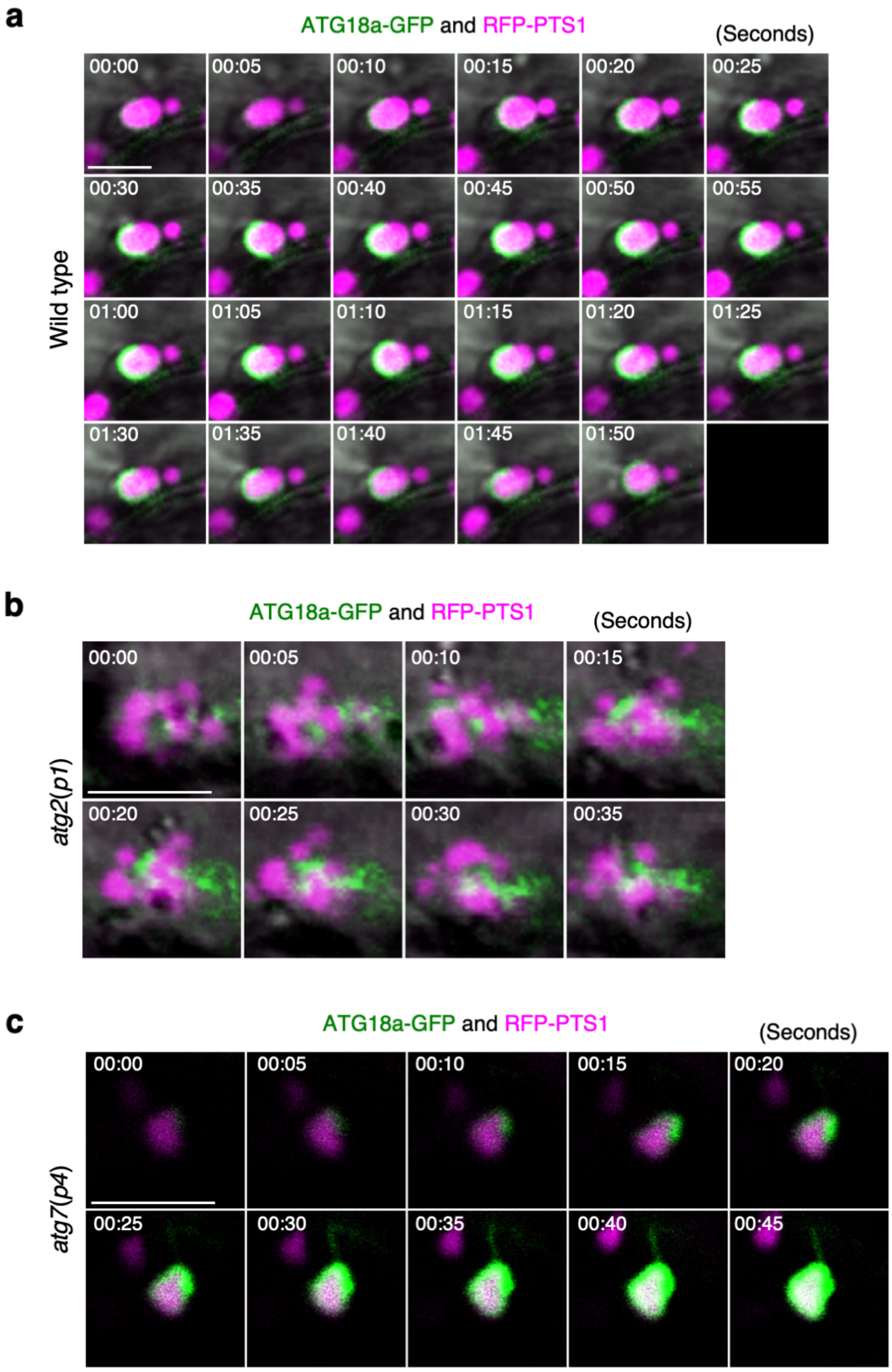

Supplementary Figure 10. Time-lapse analyses of ATG18a-GFP in wild type, *atg2,* and *atg7*

a–c, Time-lapse images of peroxisome (magenta) and ATG18a-GFP (green) in leaf mesophyll cell from wild -type (**a**), *atg2*(*p1*) (**b**), and *atg7*(*p4*) plants (**c**) were obtained every 5 s. ATG18a-GFP surrounds the peroxisome in wild-type (**a**) and *atg7*(*p4*) (**c**), but not in *atg2*(*p1*) plants (**b**). Three-week-old plants cultured on an agar plate containing ½ MS with 1% sucrose under normal-intensity (100 µmol m^{-2} s^{-1}) white-light conditions were used. The representative images in (**a–c**) show a summary of ten independent experiments. Scale bars are 2 µm in (**a**) and 5 µm in (**b**, **c**).

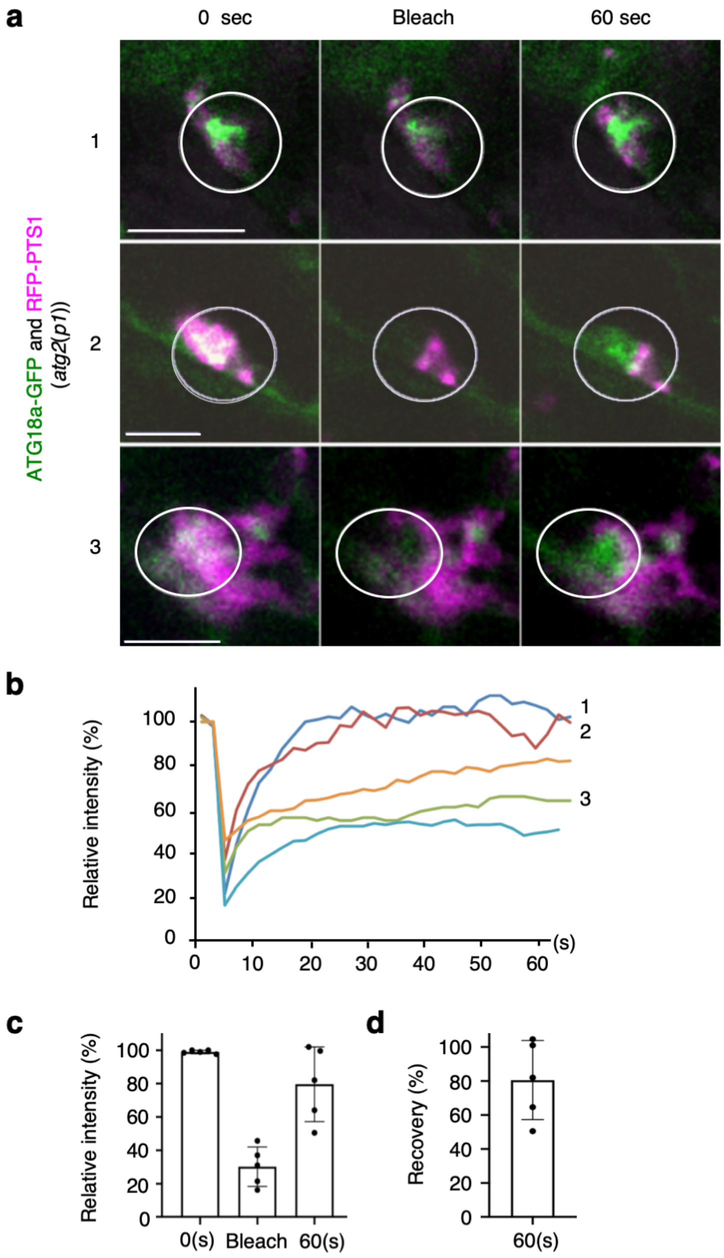

Supplementary Figure 11. FRAP analysis for ATG18a-GFP targeting peroxisome aggregates in *atg2*(*p1*)
a, Three representative images (1–3) of fluorescence recovery after photobleaching (FRAP) analysis of ATG18a-GFP in *atg2*(*p1*) plants. The images were obtained from the surface region of leaf mesophyll cells of three-week-old plants cultured on an agar plate containing ½ MS with 1% sucrose under normal-intensity (100 μmol m^{-2} s^{-1}) white-light conditions. The images shown are before (0 s), just after (Bleach), and 60 s after the bleaching. Scale bars, 5 μm. The representative images show a summary of at least five independent experiments. **b**, Kinetic changes of GFP fluorescence. Five individuals following FRAP analyses are shown in different colours. The numbers 1–3 correspond to those in (**a**). 1; blue line, 2; orange line, and 3; light blue line. **c**, Relative GFP fluorescence in the five FRAP analyses in (**b**). **d**, Fluorescence recovery of GFP after bleaching was calculated from (**c**). The error bars indicate mean ± standard deviation (*n* = 5 biologically independent peroxisome aggregates) in (**c**, **d**).

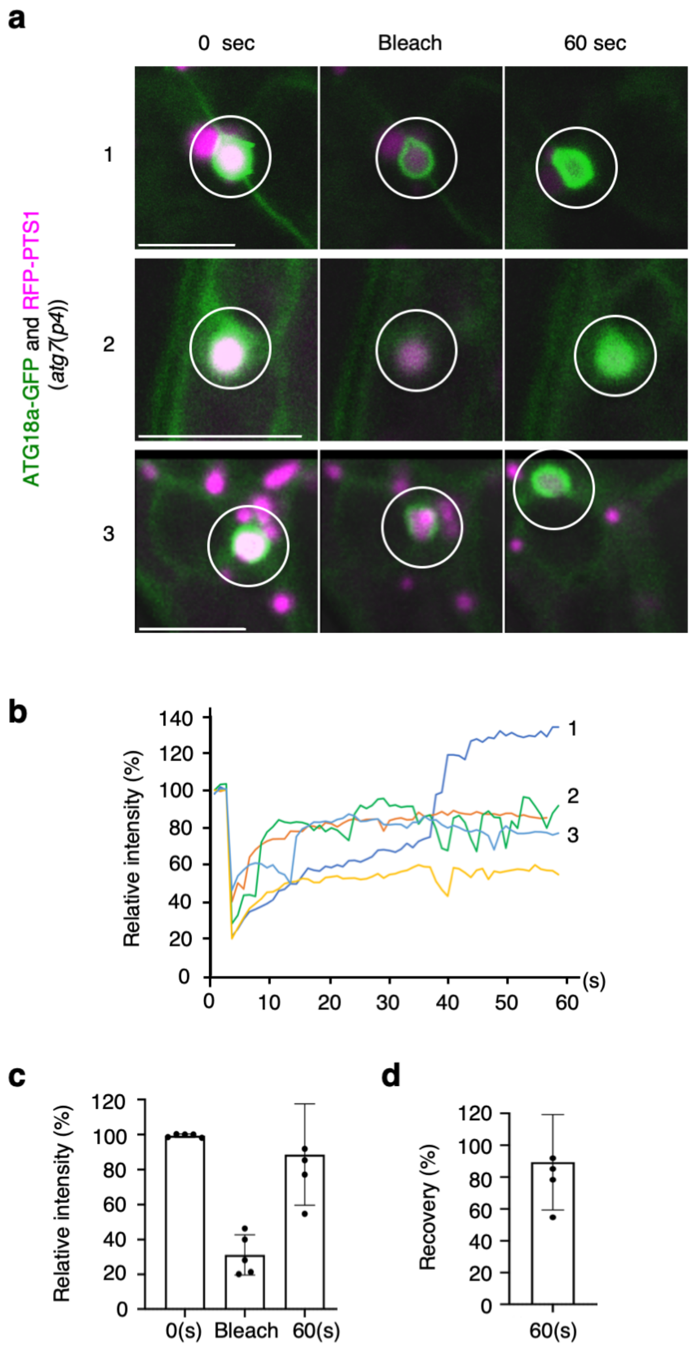

Supplementary Figure 12. FRAP analysis for ATG18a-GFP targeting peroxisome aggregates in *atg7*(*p4*)
a, Three representative images (1–3) of fluorescence recovery after photobleaching (FRAP) analysis of ATG18a-GFP in *atg7*(*p4*) plants. The images were obtained from the surface region of leaf mesophyll cells of three-week-old plants cultured on an agar plate containing ½ MS with 1% sucrose under normal-intensity (100 μmol m^{-2} s^{-1}) white-light conditions. The images shown are before (0 s), just after (Bleach), and 60 s after the bleaching. Scale bars, 5 μm. The representative images show a summary of at least five independent experiments. **b**, Kinetic changes of GFP fluorescence. Five individuals following FRAP analyses are shown in different colours. The numbers 1–3 correspond to those in (**a**). 1; blue line, 2; orange line, and 3; light blue line. **c**, Relative GFP fluorescence in the five FRAP analyses in (**b**). **d**, Fluorescence recovery of GFP after

bleaching was calculated from (**c**). The error bars indicate mean ± standard deviation (n = 5 biologically independent peroxisomes enveloped with ATG18a-GFP) in (**c**, **d**).

a

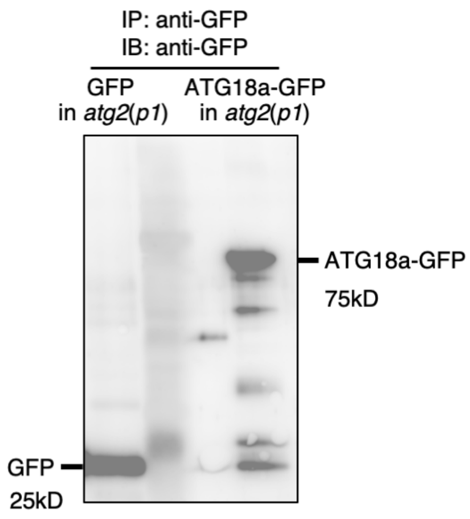

b

Number of proteins in data bases and immunoprecipitation, and recovery ratio.

	PPDB [a]	SUBA4 [a]	IP [b]	IP/PPDB (%) [c]	IP/SUBA4 (%) [c]
Chloroplast	2433	3281	15	0.62	0.46
Mitochondrion	567	2170	2	0.35	0.09
Peroxisome	228	292	8	3.51	2.74

[a] Number of proteins in the organelles indicated in the data bases (PPDB and SUBA4). [b] Number of proteins identified in immunoprecipitation of ATG18a-GFP (IP). [c] Recovery ratio calculated from the numbers.

Supplementary Figure 13. Mass spectrometry analysis to identify ATG18a-GFP interacting proteins
a, Immunoblotting of samples for MS analysis with anti-GFP antibody. **b**, Number of proteins in databases, immunoprecipitation, and recovery ratio. GFP and ATG18a-GFP bans are indicated at 25 kDa and 75 kDa, respectively. The representative images show a summary of three independent experiments.

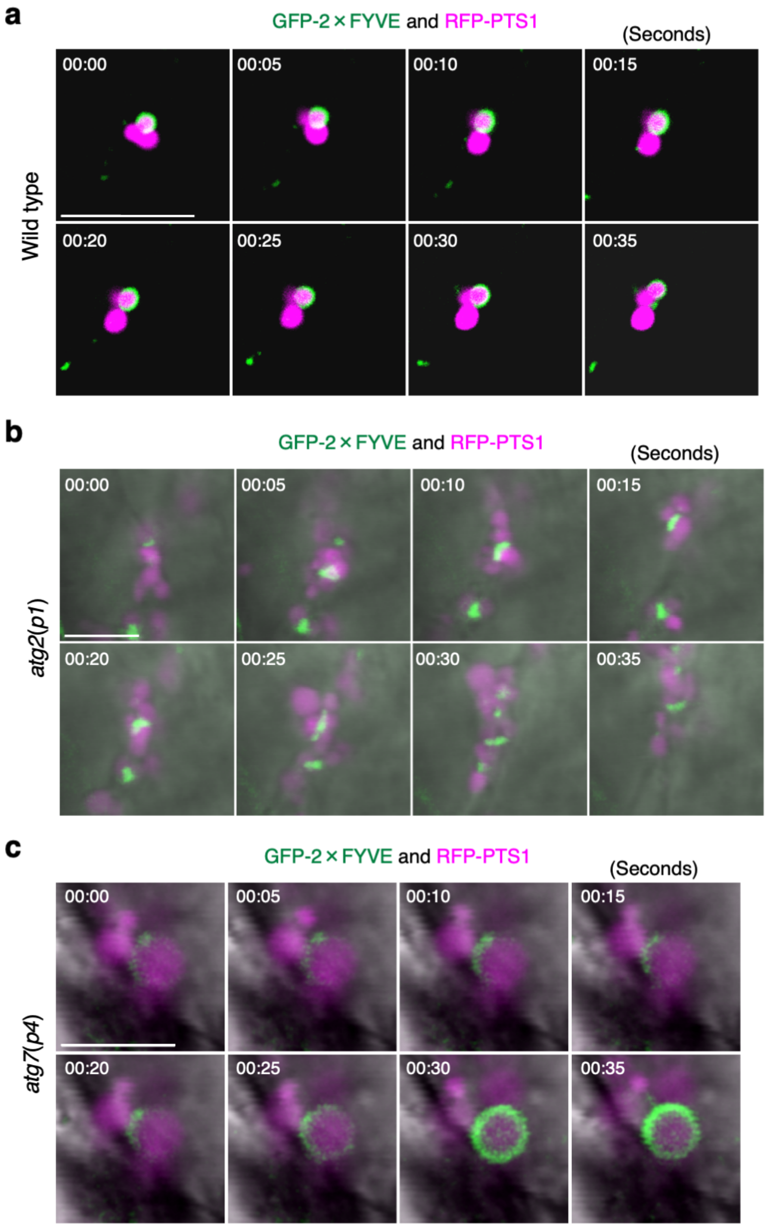

Supplementary Figure 14. Time-lapse analyses of GFP-2×FYVE in wild-type, *atg2*, and *atg7* cells

a–c, Time-lapse images of peroxisome (RFP-PTS1, magenta) and GFP-2×FYVE (green) in leaf mesophyll cells from wild-type (**a**), *atg2*(*p1*) (**b**), and *atg7*(*p4*) plants (**c**) were obtained every 5 s. GFP-2×FYVE surrounds the peroxisome in wild type (**a**) and *atg7*(*p4*) (**c**), but not in *atg2*(*p1*) plants (**b**). Three-week-old plants cultured on an agar plate containing ½ MS with 1% sucrose under normal-intensity (100 µmol m^{-2} s^{-1}) white-light conditions were used. The representative images in (**a–c**) show a summary of ten independent experiments. Scale bars, 5 µm in (**a**, **b**, and **c**).

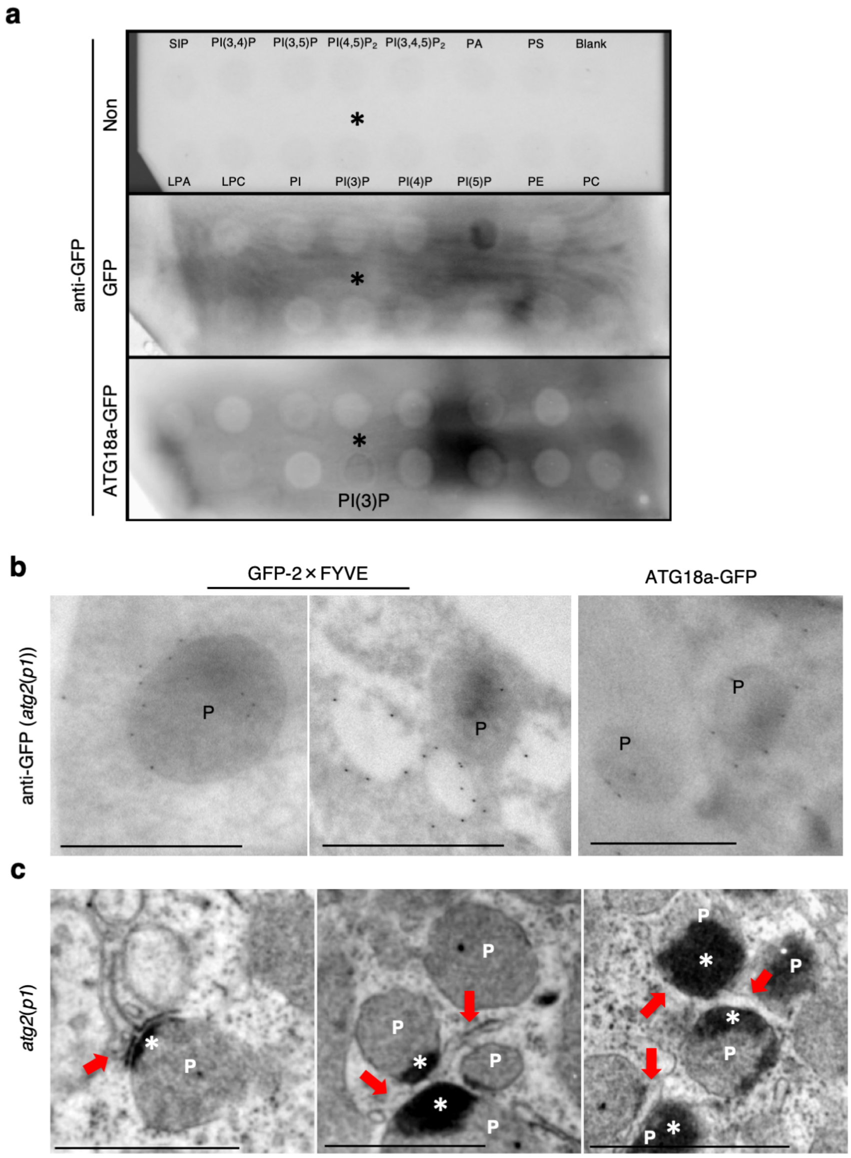

Supplementary Figure 15. Lipid-binding test and electron microscopy analyses show that ATG18a-GFP targets PtdIns3P formed on peroxisomes in *atg2*

a, Binding of ATG18a-GFP to PtdIns3P (PI(3)P; black asterisks) on membrane lipid strips. The bound ATG18a-GFP and GFP were detected following immunoblotting with an anti-GFP antibody. Each spot displays Sphingosine 1-phosphate, S1P; PtdIns(3,4)P_2, PI(3,4)P_2; PtdIns(3,5)P_2, PI(3,5)P_2; PtdIns(4,5)P_2, PI(4,5)P_2; PtdIns(3,4,5)P_3, PI(3,4,5)P_3; Phosphatidic acid: PA; PtdSer, PS; Lysophosphatidic acid, LPA; Lysophosphocholine, LPC; PtdIns4P, PI(4)P; PtdIns5P, PI(5)P; PtdEtn, PE; and PedCho, PC. **b**, Immunoelectron microscopy analysis of *atg2*(*p1*) cells expressing *GFP-2×FYVE* (left and middle) and *ATG18a-GFP* (right) using an antibody against GFP. Black dots show GFP-2×FYVE and ATG18a-GFP on peroxisomes (P), which contain high-density regions. Note that GFP-2×FYVE localises on both peroxisomes and PAS-like structures formed around peroxisomes, whereas ATG18a-GFP localises on the periphery of peroxisomes. Scale bars, 1 µm. **c**, ER and the PAS-like structures (red arrows) are close to the high-dense area (white asterisks) in peroxisomes of *atg2*(*p1*). Scale bars, 2 µm. Three-week-old plants cultured on an agar plate containing ½ MS with 1% sucrose under normal-intensity (100 µmol m^{-2} s^{-1}) white-light conditions were used. The representative images in (**b**, **c**) show a summary of at least five repeated experiments.

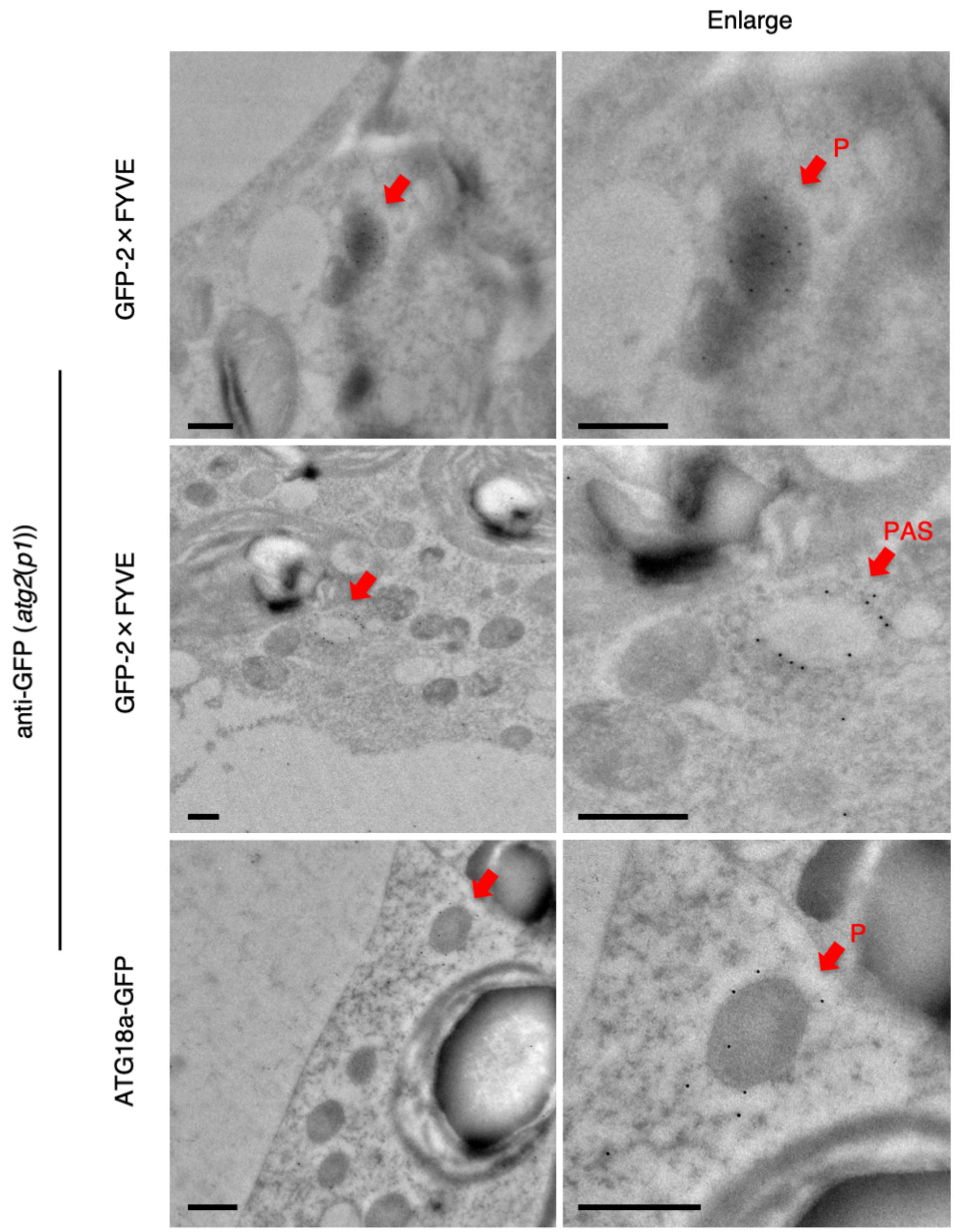

Supplementary Figure 16. Immunoelectron microscopy analysis of GFP-2×FYVE and ATG18a-GFP localisation in *atg2*

Immunoelectron microscopy analysis using an anti-GFP antibody in *atg2*(*p1*) cells expressing *GFP-2×FYVE* (top and middle) and *ATG18a-GFP* (bottom). Large-field images (left column) and enlarged images (right column) are shown. Peroxisomes (P) and PAS-like structures (PAS) are indicated with red arrows. Black dots show the localisation of GFP-2×FYVE or ATG18a-GFP, which are detected on peroxisomes containing high-density regions. Note that GFP-2×FYVE localised on both peroxisomes and PAS-like structures formed around peroxisomes, while ATG18a-GFP localised on the periphery of peroxisomes. Scale bars, 1 μm. The representative images show a summary of five repeated experiments.

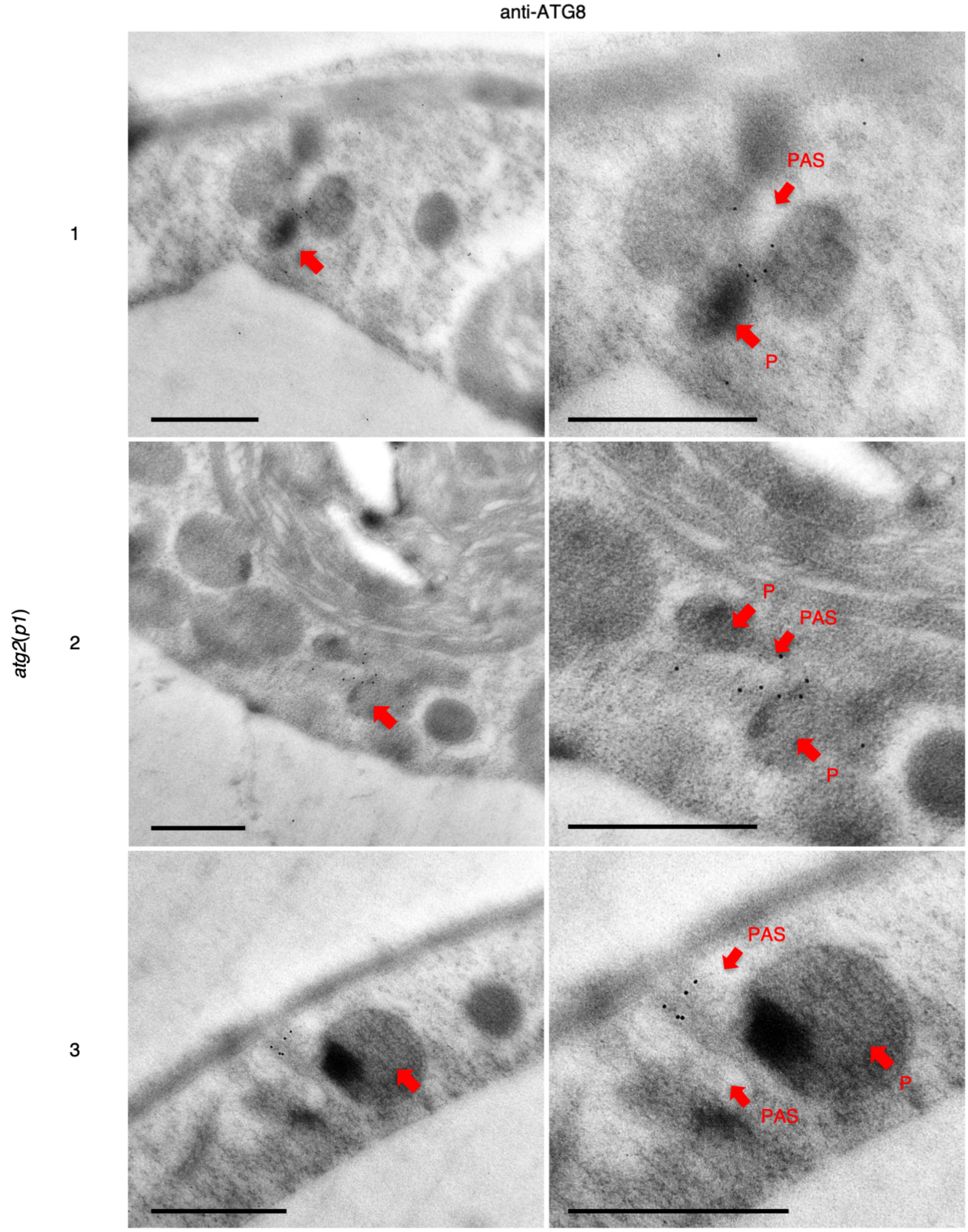

Supplementary Figure 17. Immunoelectron microscopy analysis of ATG8 on peroxisomes in *atg2* Immunoelectron microscopy analysis of *atg2*(*p1*) cells using an anti-ATG8 antibody. Three representative images are shown in large-field (left column) and enlarged (right column) views. Peroxisomes (P) and PAS-like structures (PAS) are indicated with red arrows. Black dots show the localization of ATG8. Note that ATG8 localised on both peroxisome containing high-density regions and PAS formed around them. Scale bars, 1 µm. The representative images show a summary of five repeated experiments.

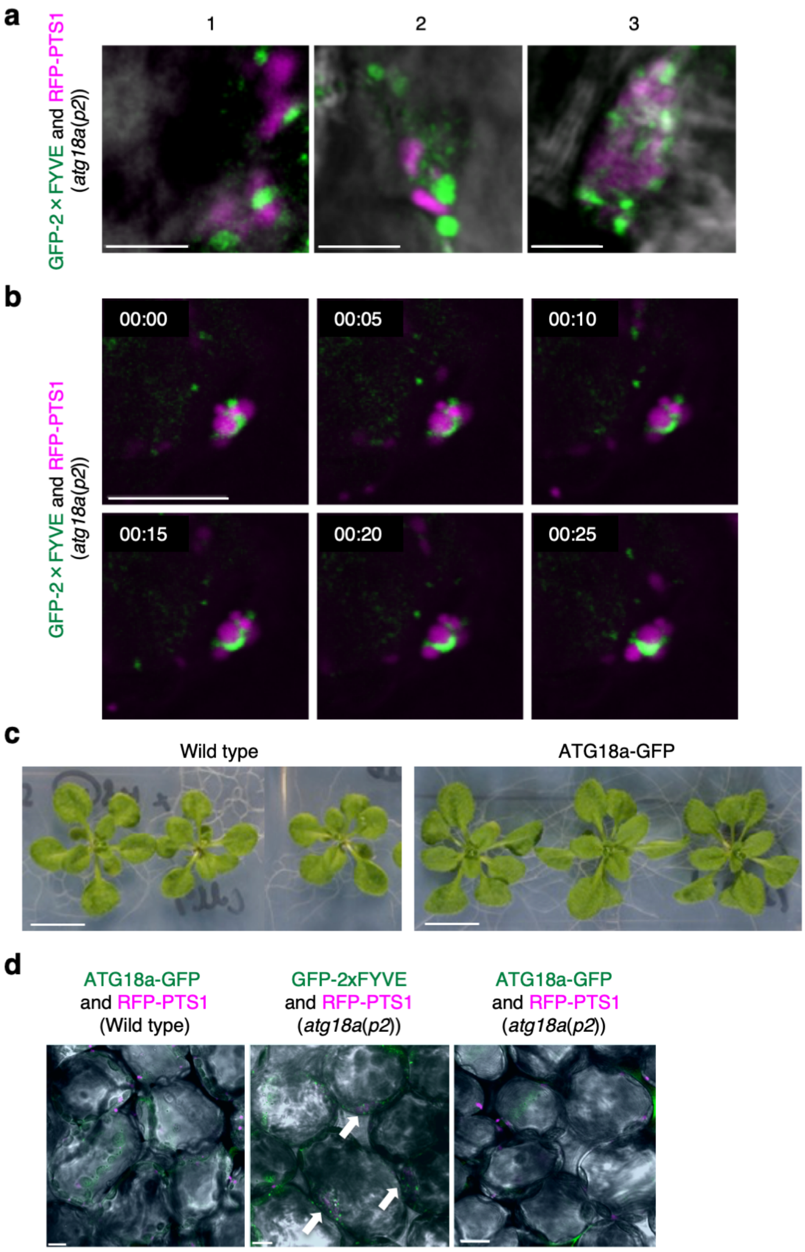

Supplementary Figure 18. ATG18a-GFP complements the mutant phenotype of *peup2/atg18a*

a, Three images of peroxisomes (RFP-PTS1, magenta) and GFP-2×FYVE (green) in *atg18a*(*p2*) cells. The images are obtained from the surface region of leaf mesophyll cells of three-week-old plants cultured on an agar plate containing ½ MS with 1% sucrose under normal-intensity (100 µmol m^{-2} s^{-1}) white-light conditions. Scale bars, 5 µm. The representative images in (**a**, **b**) show a summary of five repeated experiments. **b**, Time-lapse images of peroxisomes and GFP-2×FYVE in *atg18a*(*p2*) cells were taken every 5 s. Note that GFP-2×FYVE targeted peroxisome aggregates in ATG18a-deficient *atg18a*(*p2*) cells. Scale bar, 5 µm. **c,** Growth of wild-type (Col-0) and transgenic plants expressing *ATG18a-GFP* (*n* = 3 technically independent replicates). Scale bar, 1 cm. **d**, Merged images show peroxisomes (RFP-PTS1, magenta), ATG18a-GFP or GFP-2×FYVE (green), and chloroplasts (grey globular structures) in cells from wild-type or *atg18a*(*p2*) plants. Peroxisome aggregates are indicated with white arrows. Note that *atg18a*(*p2*) cells expressing *ATG18a-GFP* display similar peroxisomes as those observed in wild-type cells. Scale bars, 10 µm. The representative images are selected from five independent experiments.

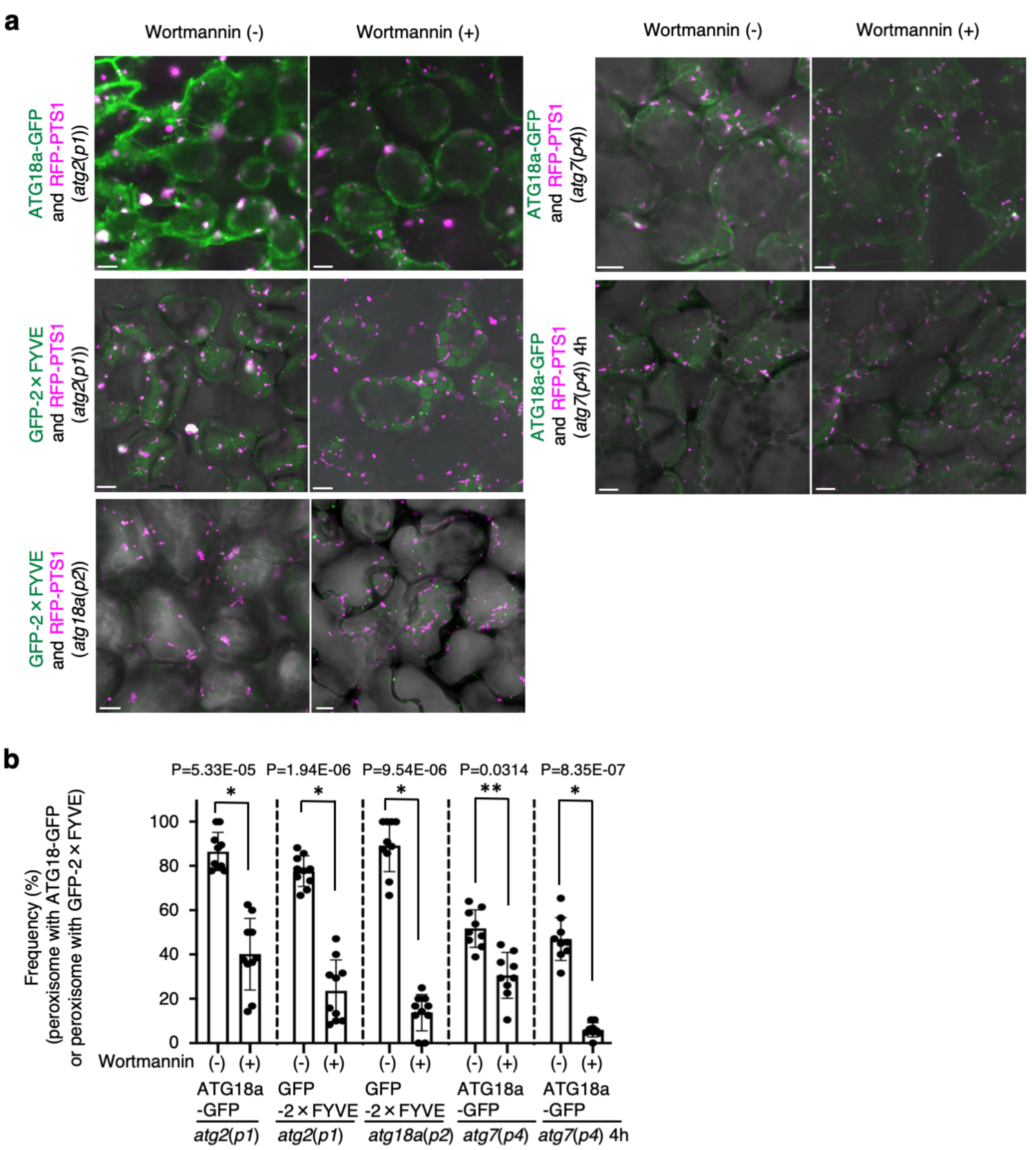

Supplementary Figure 19. Wortmannin disturbs the accumulation of ATG18a-GFP and GFP-2×FYVE on peroxisomes in leaf cells of *atg2, atg7,* and *atg18a* plants

a, CLSM images of leaf mesophyll cells of *atg2*(*p1*) expressing ATG18a-GFP (upper left) or GFP-2×FYVE (middle left), *atg18a*(*p2*) expressing GFP-2×FYVE (lower left), and *atg7*(*p4*) expressing ATG18a-GFP (upper right) plants treated with (+) or without (-) 20 µM wortmannin for 2 h under normal-intensity (100 µmol m^{-2} s^{-1}) white-light conditions. For *atg7*(*p4*) expressing ATG18a-GFP, 4 h wortmannin treatment was also tested (lower right). These plants were also expressing peroxisome marker RFP-PTS1. Three-week-old plants cultured on an agar plate containing ½ MS with 1% sucrose were used. Scale bars, 10 µm. **b,** Ratio of peroxisome aggregate bound by ATG18a-GFP or GFP-2×FYVE to total peroxisome aggregate in leaf mesophyll cells before and after the treatment with wortmannin. The error bars indicate mean ± standard deviation and asterisks indicate significant differences between each plant treated with (+) or without (-) 20 µM wortmannin (*P < 0.01, two-sided Student's *t*-test) (n = 100 biologically independent peroxisome aggregates from at least nine independent experiments). The representative images in (**a**) show a summary of at least nine independent experiments.

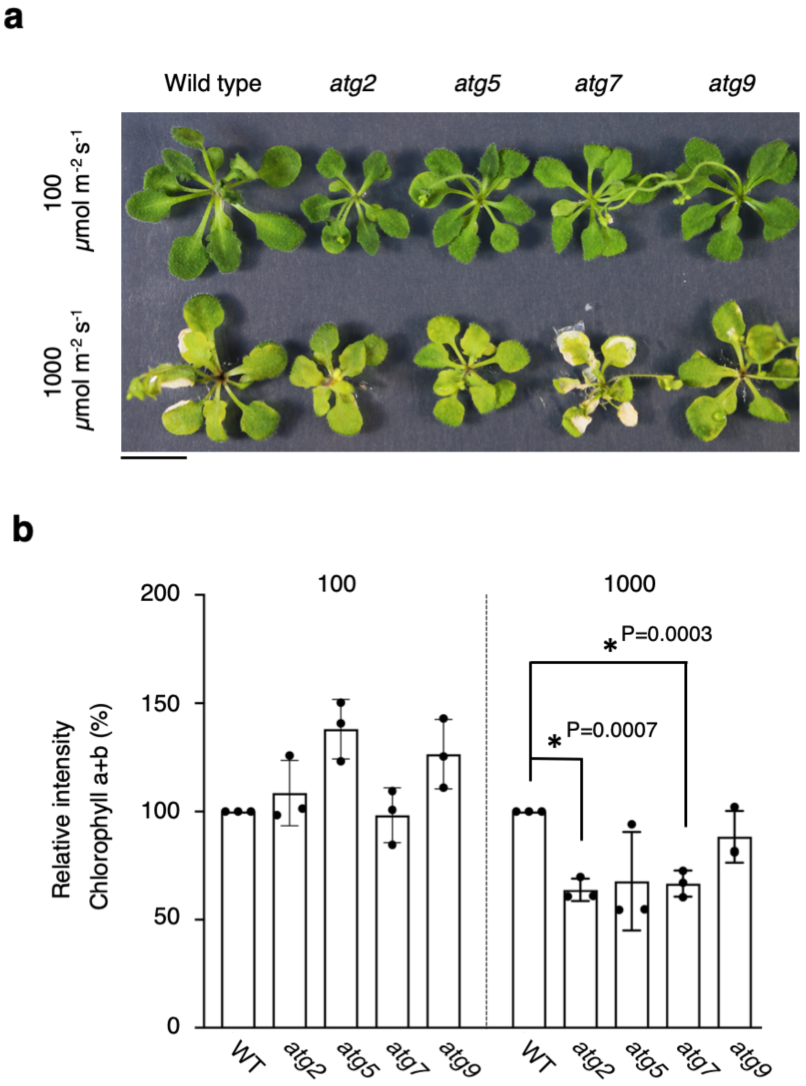

Supplementary Figure 20. Effect of high-intensity light on leaf damage and chlorophyll reduction

a, Growth analysis of wild-type, *atg2-1, atg5-1, atg7-2*, and *atg9-3* plants under low (100 µmol m^{-2} s^{-1}) and high-intensity (1000 µmol m^{-2} s^{-1}) light with an LED (blue and red light) equipment. The three-week-old plants cultured on an agar plate containing ½ MS with 1% sucrose under normal-intensity (100 µmol m^{-2} s^{-1}) white-light conditions were moved to the high-intensity light conditions described above. Scale bar, 1 cm. **b**, Relative intensity of chlorophyll a + b extracted from wild-type, *atg2, atg5, atg7*, and *atg9* leaves grown under low (100 µmol m^{-2} s^{-1}) and high-intensity (1000 µmol m^{-2} s^{-1}) light. The error bars indicate mean ± standard deviation (n = 12 biologically independent plants from three technically independent replicates) and asterisks indicate significant differences between wild type and *atg2, atg5,* or *atg7* (*$P < 0.01$, **$P < 0.05$, two-sided Student's t-test). The representative images in (**a**) show a summary of four independent experiments.

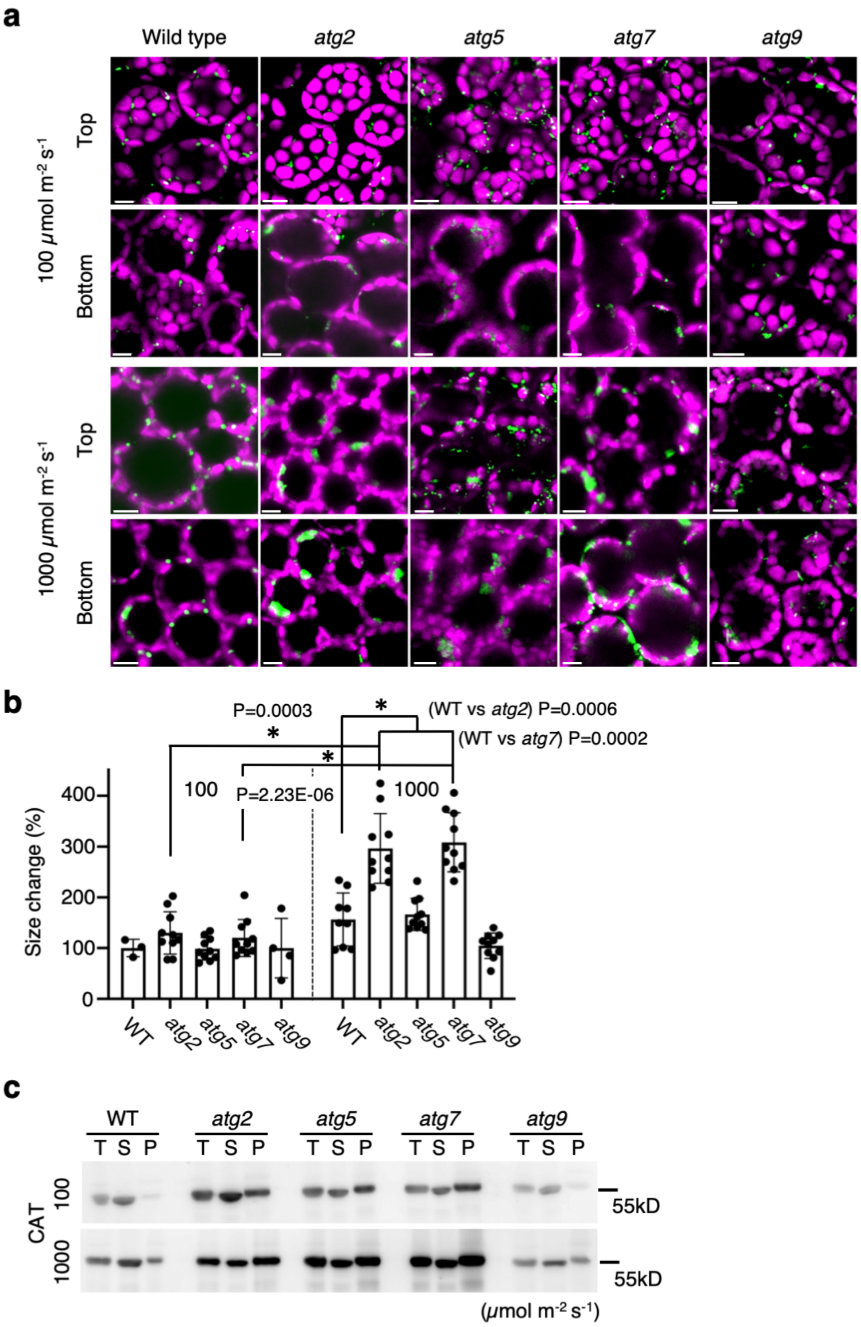

Supplementary Figure 21. Effect of high-intensity light on induction of large peroxisome aggregates, and accumulation of catalase in autophagy mutants

a, Peroxisomes (GFP-PTS1, green) and chloroplasts (chlorophyll autofluorescence, magenta) within the region from top to middle (Top) and from middle to bottom (Bottom) of leaf mesophyll cells of wild-type, *atg2, atg5, atg7*, and *atg9* plants under normal (100 μmol m^{-2} s^{-1}) and the high-intensity light (1000 μmol m^{-2} s^{-1}). Scale bars, 10 μm. The representative image show a summary of five independent experiments. Note that large peroxisome aggregates were formed in *atg2* and *atg7* under the high-intensity light. **b**, Relative size of peroxisome aggregates in (**b**) is calculated (n= 100 biologically independent leaf cells). The error bars indicate mean ± standard deviation (*n* = 10 biologically independent replicates) and asterisks indicate significant differences in peroxisome size between wild type and *atg2* or *atg7* (**P* < 0.01, two-sided Student's *t*-test). **c**, Immunoblot analysis of catalase (CAT) in total (T), supernatant (S), and pellets (P) of leaf extract. The representative images in (**c**) show a summary of three (**c**) repeated experiments.

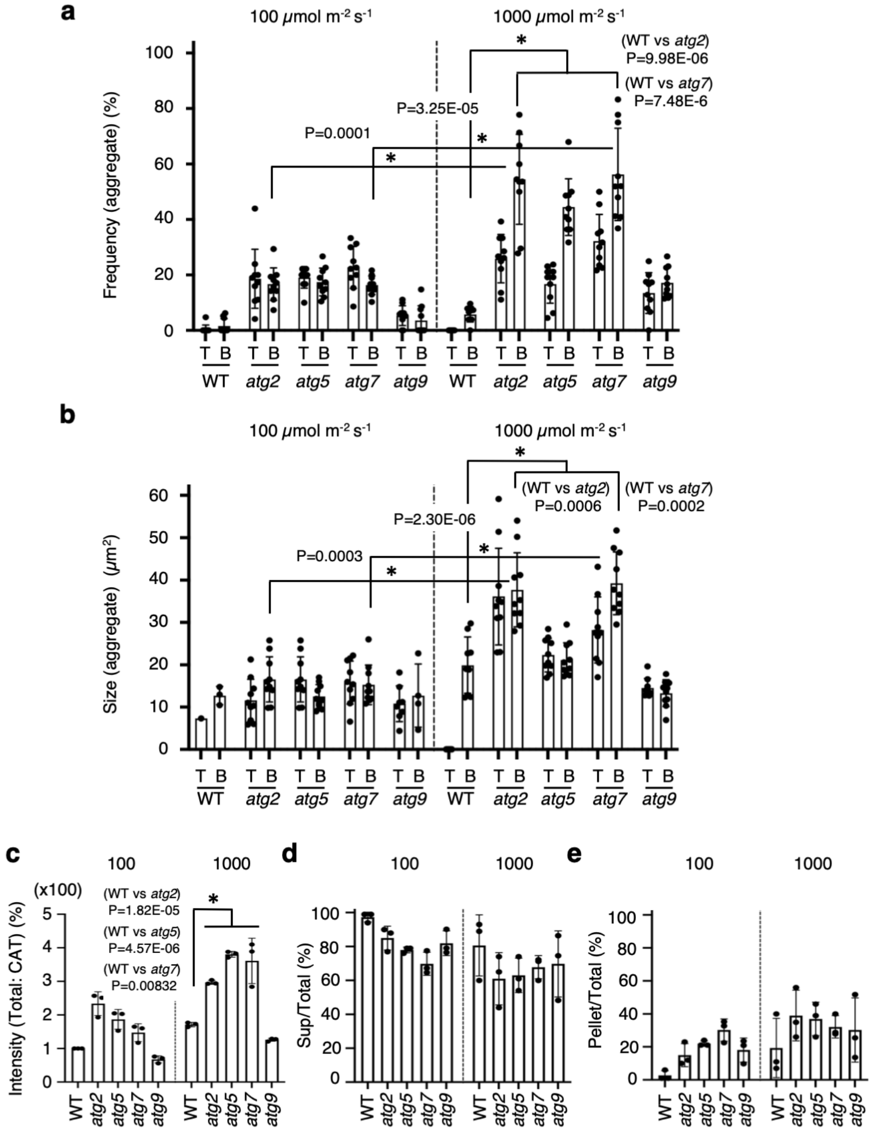

Supplementary Figure 22. Quantification of large peroxisome aggregates and catalase accumulation in wild-type, *atg2*, *atg5*, *atg7*, and *atg9* cells

a, **b**, Frequency of cells containing peroxisome aggregates (**a**) and size of peroxisome aggregates (**b**) in wild type (WT) and mutant lines corresponding to **Supplementary Fig. 21**(**a**). T, top to middle region; B, middle to bottom region of mesophyll cells. More than 100 cells were tested in (**a**). Each tested number of peroxisome aggregates for low-intensity light in (**b**): WT top (n = 6), WT bottom (n = 28), *atg2* top (n = 50), *atg2* bottom (n = 63), *atg5* top (n = 43), *atg5* bottom (n = 65), *atg7* top (n = 83), *atg7* bottom (n = 90), *atg9* top (n = 22), and *atg9* bottom (n = 18); for high-intensity light in (**b**): WT top (n = 19), WT bottom (n = 48), *atg2* top (n = 52), *atg2* bottom (n = 55), *atg5* top (n = 45), *atg5* bottom (n = 76), *atg7* top (n = 42), *atg7* bottom (n = 49), *atg9* top (n = 40), and *atg9* bottom (n = 41). The error bars indicate mean ± standard deviation (n = 10 biologically independent replicates) and asterisks indicate significant differences in the size of peroxisome aggregates between WT and *atg2* or *atg7* (*$P < 0.01$, two-sided Student's t-test). **c-e**, Signal intensity (**c**), the ratio of sup to total extraction (**d**), and pellet to total extraction (**e**) of immunoblotting analysis using the anti-CAT antibody in **Supplementary Fig. 21**(**c**) were measured using Dot Blot Analysis in ImageJ. The results of each fraction and supernatant or pellet per total are shown as graphs. The error bars indicate mean ± standard deviation and asterisks indicate significant differences between WT and *atg2* or *atg7* (*$P < 0.01$, two-sided Student's t-test). (n =3 biologically independent plants) in (**c**). The calculation is shown in Data source file.

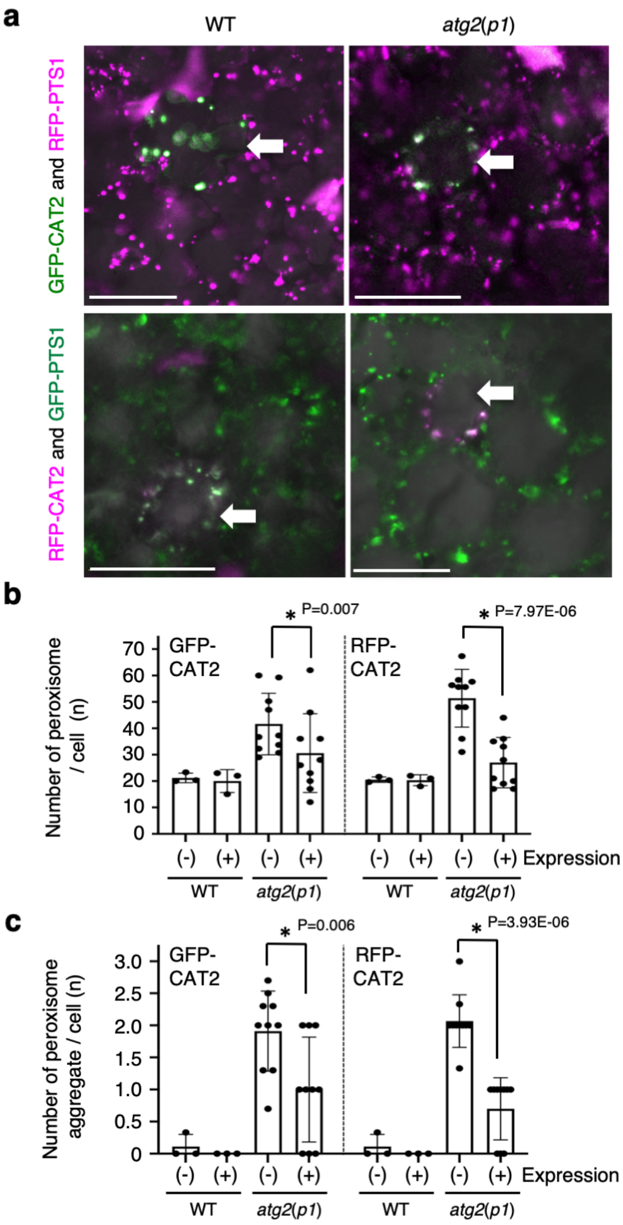

Supplementary Figure 23. Suppression of peroxisome mutant phenotype in *atg2* following ectopic expression of Catalase

a, CLSM images of peroxisomes (RFP-PTS1 in upper panels or GFP-PTS1 in lower panels) in leaf mesophyll cells of wild type (WT) and *atg2*(*p1*) expressing GFP-CAT2 or RFP-CAT2 after adaptation to high-intensity (500 μmol m^{-2} s^{-1}) light produced using an LED equipment (blue and red light) for 8 h. Three-week-old plants cultured on an agar plate containing ½ MS with 1% sucrose. White arrows indicate the cells expressing GFP-CAT2 or RFP-CAT2, which are transiently expressed by a particle bombardment. Scale bars, 10 μm. **b, c,** The number of peroxisomes (**b**) and peroxisome aggregates (**c**) in leaf mesophyll cells of WT and *atg2*(*p1*) with (+) or without (-) expression of GFP-CAT2 or RFP-CAT2. The error bars indicate mean ± standard deviation and asterisks indicate significant differences between *atg2*(*p1*) and *atg2*(*p1*) expressing GFP(RFP)-CAT2 (**P* < 0.01, two-sided Student's *t*-test) (*n* = 10 biologically independent cells from eight independent leaves) in (**b, c**). The representative images show a summary of at least ten biologically independent cells.

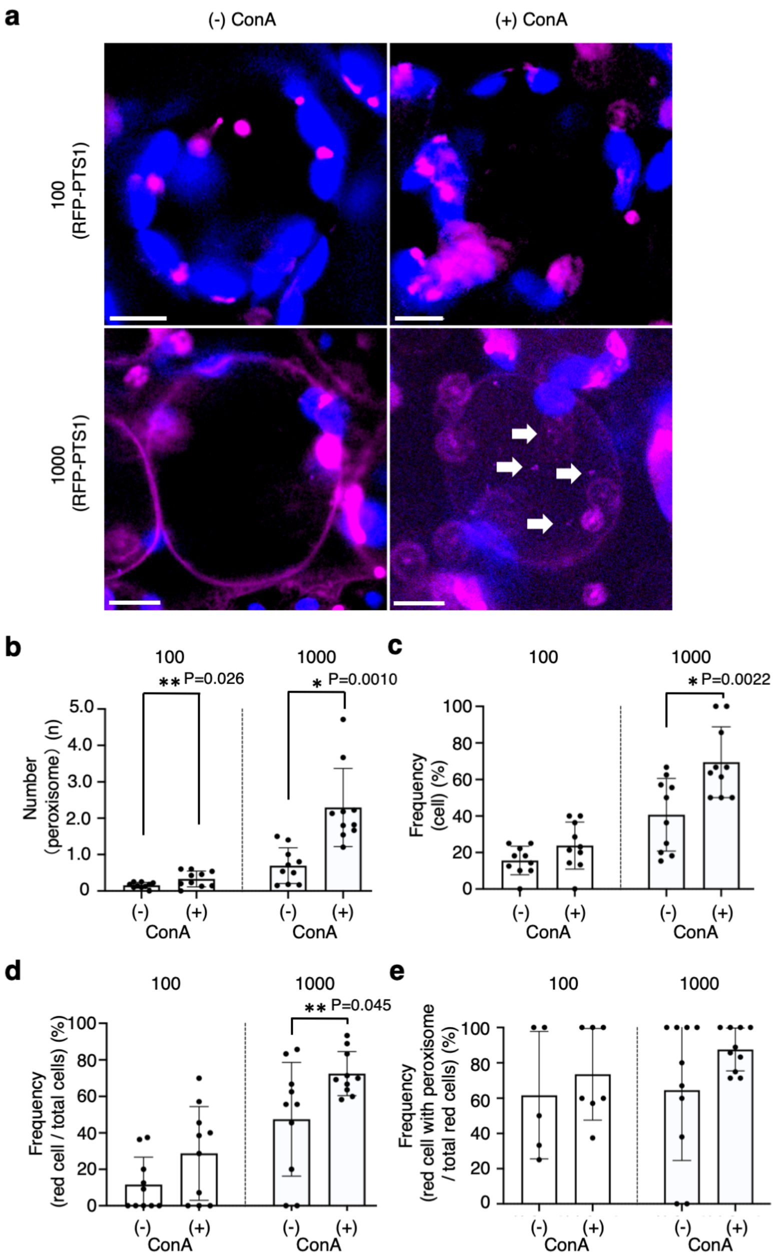

Supplementary Figure 24. Effect of concanamycin A on the peroxisome degradation

a, Representative images of peroxisomes (RFP-PTS1, magenta) and chloroplast (blue) in leaf mesophyll cells of three-week-old wild-type plants treated with (+) or without (-) concanamycin A (ConA) under 100 and 1000 μmol m^{-2} s^{-1} light, which are the same light conditions as in Figures 4-7. Scale bars, 5 μm. **b**, The average number of peroxisomes in a vacuole**.** **c**, Frequency of cells containing peroxisomes inside the vacuole. d, Frequency of cells containing red-coloured vacuoles to the total number of cells. **e,** Frequency of cells with red-coloured vacuoles containing peroxisome to the total number of cells with red-coloured vacuoles. The error bars indicate mean ± standard deviation (n = 10 biologically independent replicates) in (**b-e**). Asterisks indicate significant differences between plants treated with (+) or without (-) ConA (*P < 0.01, **P < 0.05) in two-sided Student's t-test (**b**–**e**). The representative images in (**a**) show a summary of at least ten biologically independent leaf cells from three independent experiments.

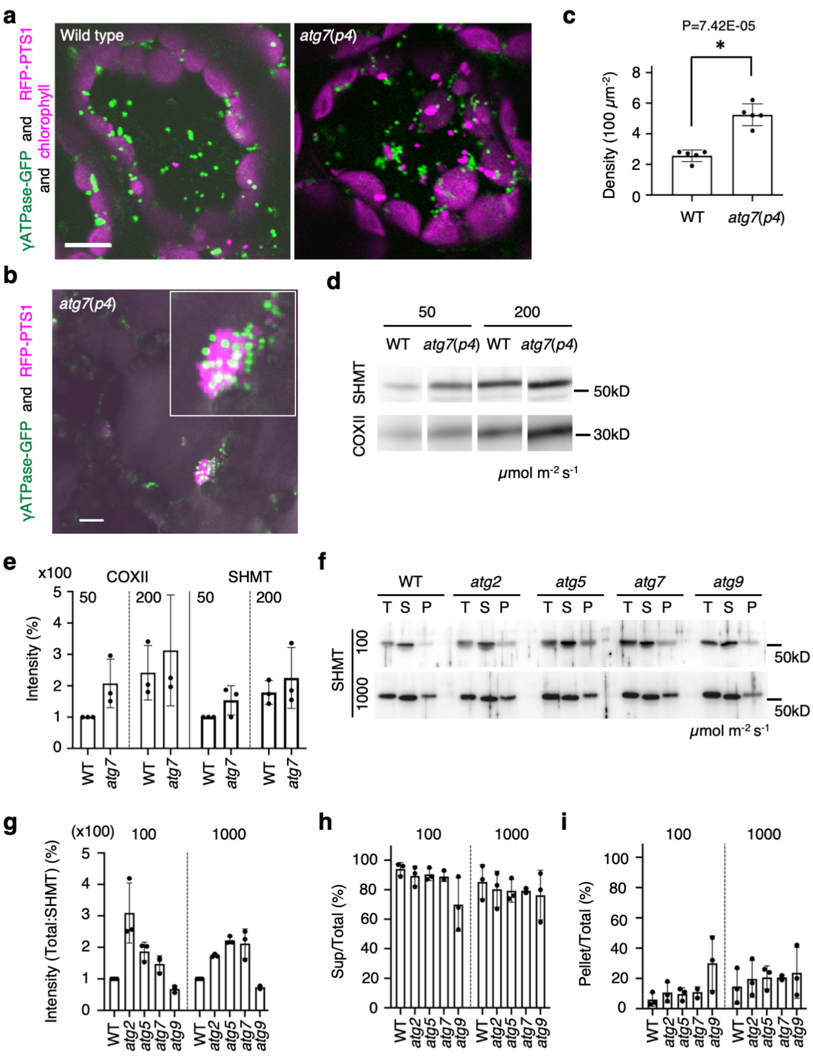

Supplementary Figure 25. Mitochondrial degradation in *atg7* is slightly impaired in light

a, Mitochondria (γATPase-GFP, green), peroxisomes (RFP-PTS1, magenta), and chloroplasts (chlorophyll autofluorescence, magenta) in the surface to middle depth area of leaf mesophyll cells from wild-type and *atg7*(*p4*) plants under normal-intensity (100 μmol m^{-2} s^{-1}) white-light condition. Three-week-old plants cultured on an agar plate containing ½ MS with 1% sucrose under normal-intensity (100 μmol m^{-2} s^{-1}) white-light conditions were used. **b**, Mitochondria are observed in the peroxisome aggregate contacting chloroplast (left). Scale bars in (**a**, **b**), 5 μm. The representative images show a summary of five independent experiments. **c**, Mitochondria density in (**a**). Data were collected from more than 100 cells imaged using CLSM. The error bars indicate mean ± standard deviation and asterisks indicate significant differences between WT and *atg7*(*p4*) (**P* < 0.01, two-sided Student's *t*-test). **d, e**, Immunoblotting analysis of leaf extract from *atg7*(*p4*) plants adapted to 50 and 200 μmol m^{-2} s^{-1} light using anti-serine hydroxymethyltransferase (SHMT) as well as anti-cytochrome c oxidase 2 (COXII) antibodies (**d**), and the relative signal intensity (**e**). **f–i,** Immunoblotting analysis of leaf-extract fractionations (total, T; supernatant, S; and pellet, P) from wild-type and *atg* mutant (*atg2-1*, *atg-5-1*, *atg7-2*, and *atg9*-3) plants adapted to 100 and 1000 μmol m^{-2} s^{-1} light (LED, blue and red light) using an anti-SHMT antibody (**f**) and the relative signal intensity (**g**), their ratios of supernatant to total (**h**), and pellet to total (**i**). The error bars indicate mean ± standard deviation (*n* =3 biologically independent plats) in (**e**, **g–i**). The representative images in (**d**, **f**) show a summary of four repeated experiments.

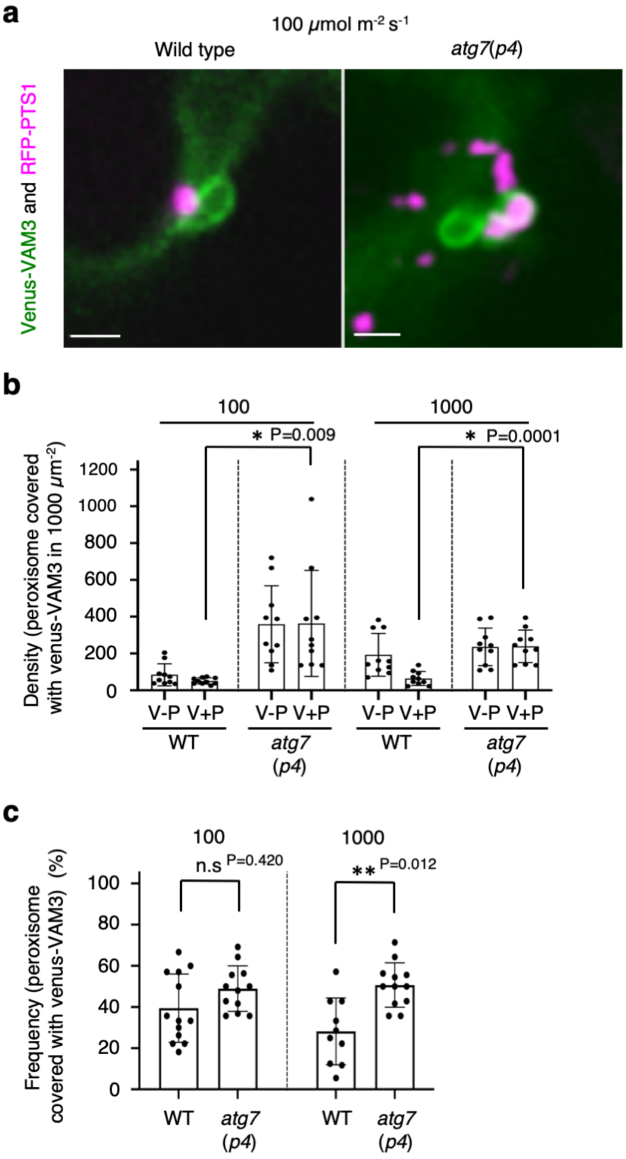

Supplementary Figure 26. Vacuole structure around peroxisomes in *atg7*

a, Vacuolar cavities surrounding peroxisomes generated from vacuolar membranes (Venus-VAM3, green) around peroxisomes (RFP-PTS1, magenta) in leaf mesophyll cells of wild-type and *atg7*(*p4*) plants. The images were obtained as described for Figs. 4–7 from the three-week-old plants grown under normal-intensity (100 µmol m^{-2} s^{-1}) white-light conditions. Plants were moved to both 100 and 1000 µmol m^{-2} s^{-1} produced using an LED equipment (blue and red light). Scale bars, 2 µm. The representative images show a summary of five repeated experiments. **b**, **c**, Density of the vacuolar cavities with (v + P) or without (v - P) peroxisomes in 1 mm^{-2} (**b**) and the frequency of the vacuolar cavities with peroxisomes in wild-type and *atg7*(*p4*) plants (**c**) under low (100 µmol m^{-2} s^{-1}) as well as high-intensity (1000 µmol m^{-2} s^{-1}) light conditions. The error bars indicate mean ± standard deviation (n = 10 biologically independent replicates). Asterisks indicate significant differences (*$P < 0.01$, **$P < 0.05$, and n.s. indicates not significant differences ($P > 0.05$) in two-sided Student's t-test (**b**, **c**).

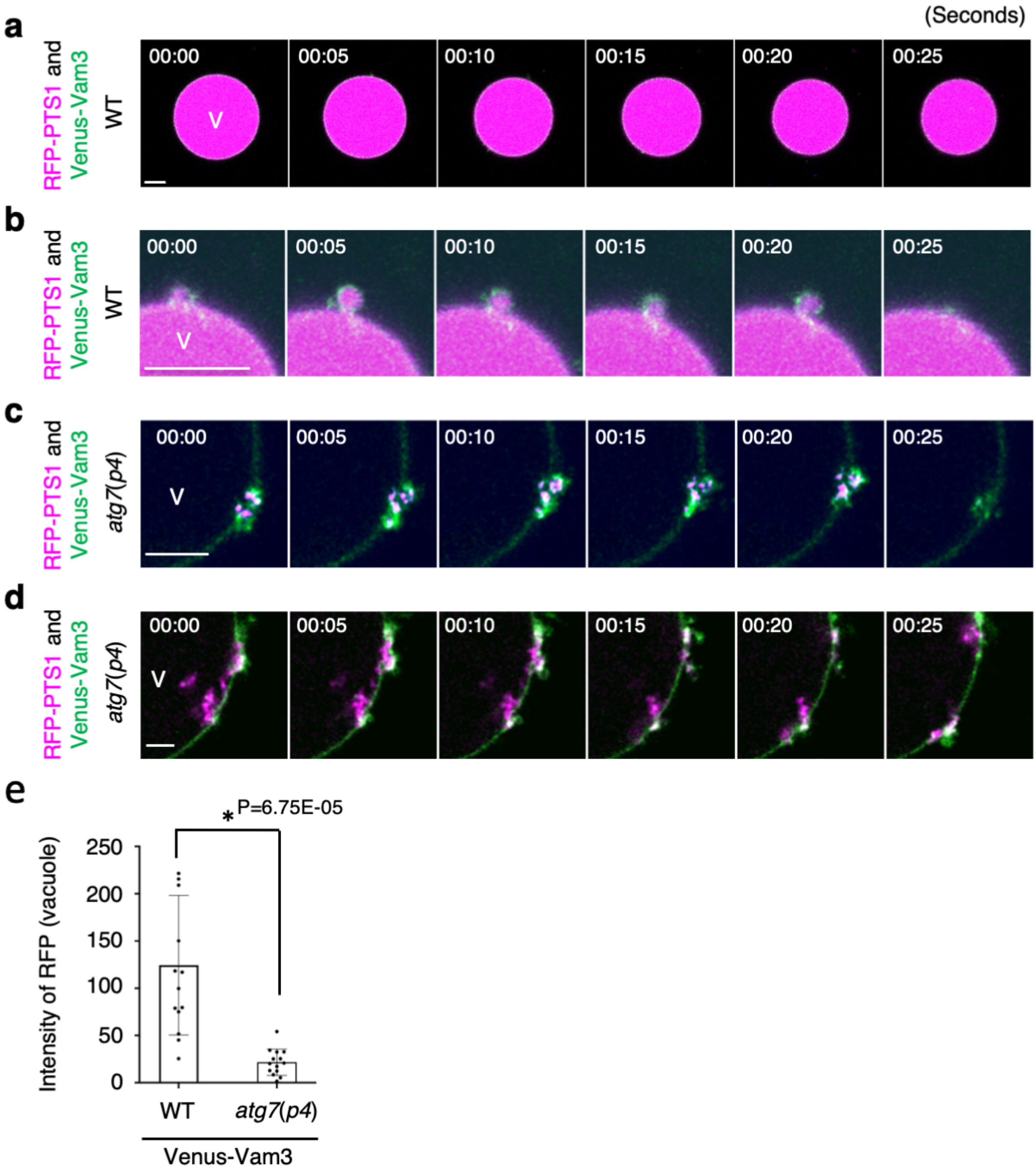

Supplementary Figure 27. Time-lapse images of peroxisomes on the surface of the isolated vacuole
a-e, Time-lapse images of vacuoles (V) isolated from leaf cells of wild-type (**a**, **b**) and *atg7*(*p4*) plants (**c**, **d**) expressing RFP-PTS1 (magenta) and Venus-VAM3 (green). The vacuoles were isolated via protoplasts derived from the leaf sections after the high-intensity (500 µmol m^{-2} s^{-1}) light irradiation using an LED equipment (blue and red light) for 5 h. Scale bars, 5 µm. **e**, Intensity of RFP fluorescence in the isolated vacuoles. The error bars indicate mean ± standard deviation ($n = 15$ biologically independent isolated vacuoles). Asterisks indicate significant differences (*$P < 0.01$) in two-sided Student's *t*-test. The representative images in (**a**–**d**) show a summary of five repeated experiments.

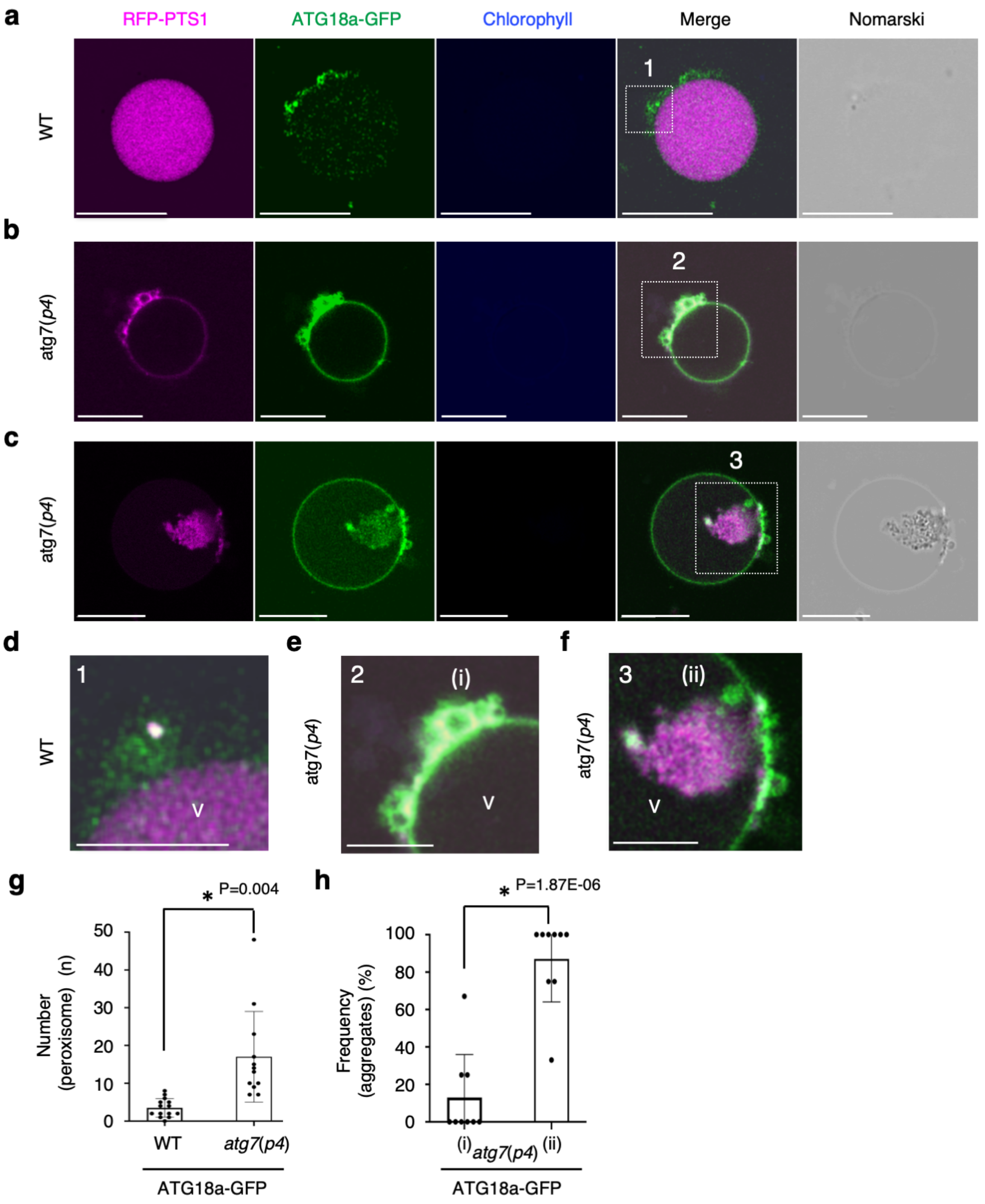

Supplementary Figure 28. Peroxisome aggregates with ATG18a-GFP are accumulated on the surface of isolated vacuoles in the *atg7*(*p4*) mutant

a–**c**, Representative images of RFP-PTS1 (magenta), ATG18a-GFP (green), chlorophyll (blue), merge, and Nomarski in isolated vacuoles from wild-type (WT) (**a**) and *atg7*(*p4*) cells (**b**, **c**) after adaptation to high-intensity (500 µmol m^{-2} s^{-1}) light produced using an LED equipment (blue and red light) for 5 h. Scale bars, 10 µm. Plants were grown in the same condition as described in **Fig. 6.** **d**–**f**, Enlarged images of isolated vacuole (V) with peroxisomes in WT (**d**) and peroxisome aggregates in *atg7*(*p4*) (**e**, **f**). Panels 1 to 3 in **a**–**c** correspond to **d**–**f**, respectively. Scale bars, 5 µm. **g**, The number of peroxisomes on/inside vacuoles isolated from WT and *atg7*(*p4*) cells. **h**, Frequency of peroxisome aggregates remarkably covered (i) or not (ii) with ATG18a-GFP on the isolated vacuoles in (**e**) and (**f**). The error bars indicate mean ± standard deviation and asterisks indicate significant differences between WT and *atg7*(*p4*) (*$P < 0.01$, Student's *t*-test) in (**g**, **h**) (n = 12 in (**g**) and n = 9 in (**h**) biologically independent isolated vacuoles). The representative images in (**a**–**f**) show a summary of four repeated experiments.

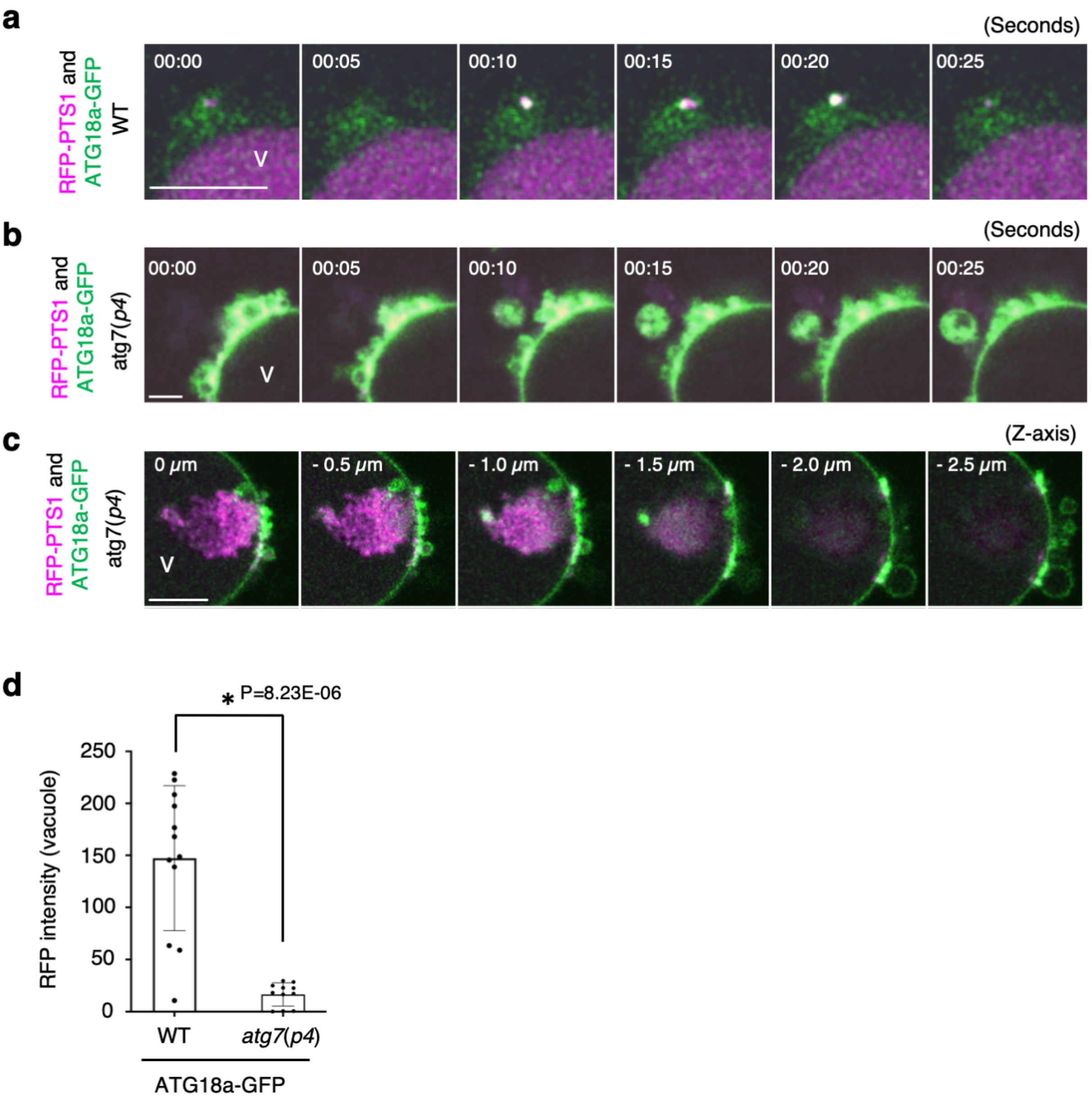

Supplementary Figure 29. Time-lapse images of peroxisomes targeted with ATG18a-GFP on the surface of the isolated vacuole

a-c, Time-lapse or z-axis images of RFP-PTS1 (magenta) and ATG18a-GFP (green) on/inside vacuoles (V) isolated from leaf cells of wild-type (**a**) and *atg7*(*p4*) plants (**b**, **c**). The images correspond to panels in **Supplementary Fig. 28**. Scale bars, 5 µm. **d,** Intensity of RFP fluorescence in the isolated vacuoles. The error bars indicate mean ± standard deviation (n = 11 biologically independent isolated vacuoles) and asterisks indicate significant differences between wild type and *atg7*(*p4*) (*$P < 0.01$, two-sided Student's *t*-test). The representative time-lapse images in (**a**–**c**) show a summary of eight repeated experiments.

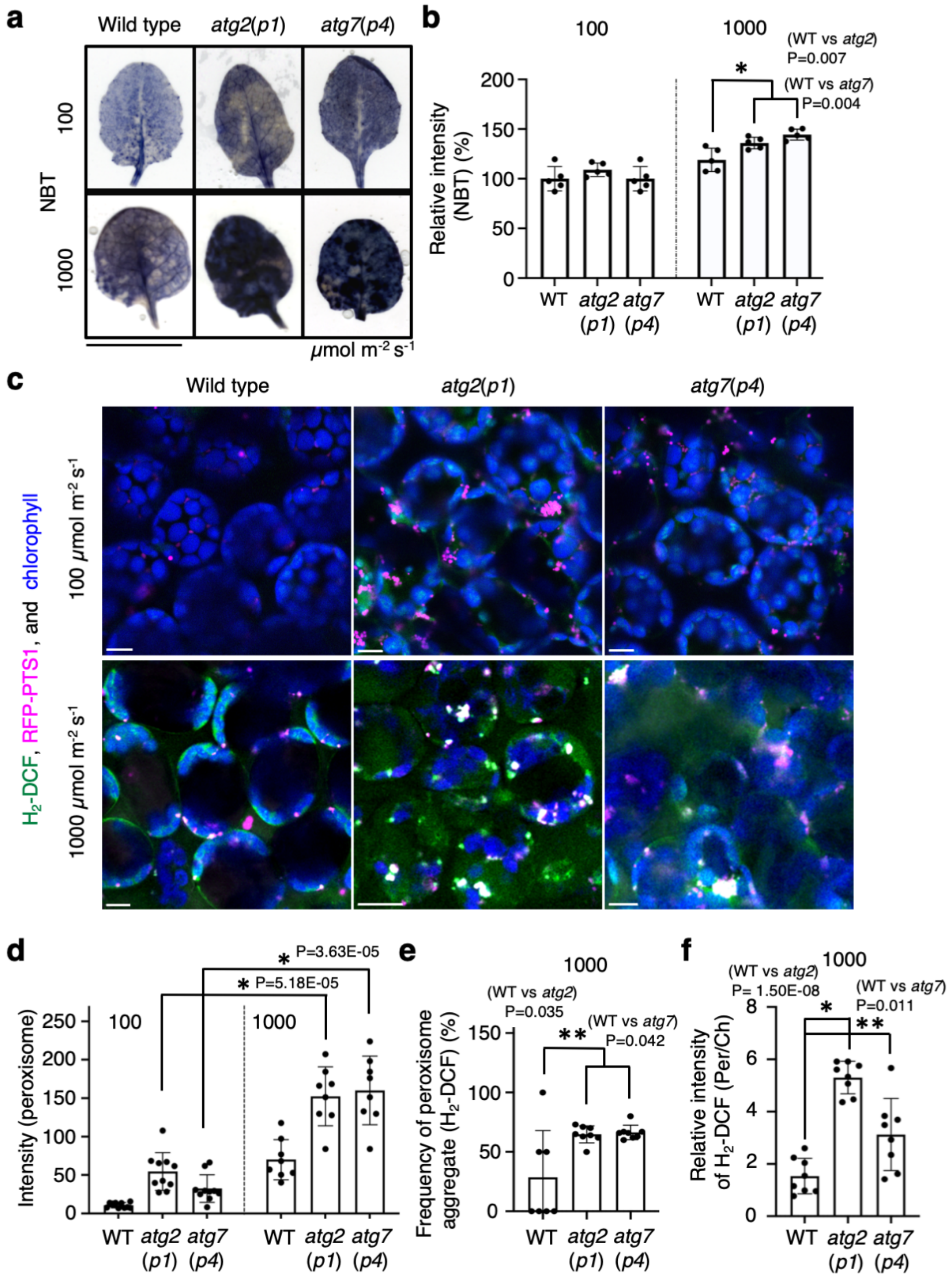

Supplementary Figure 30. High accumulation of ROS in the peroxisomes of *atg2* and *atg7* under high-intensity light

a, Nitroblue tetrazolium (NBT)-stained leaves of wild-type (WT), *atg2*(*p1*), and *atg7*(*p4*) plants after exposure to low (100 μmol m^{-2} s^{-1})- and high (1000 μmol m^{-2} s^{-1})-intensity light produced using an LED equipment (blue and red light) for 16 h. Three-week-old plants cultured on an agar plate containing ½ MS with 1% sucrose under normal-intensity (100 μmol m^{-2} s^{-1}) white-light conditions were moved to each light condition. Scale bar, 1 cm. **b**, Relative NBT intensity in leaves of WT, *atg2*(*p1*), and *atg7*(*p4*) plants. The error bars indicate mean ± standard deviation ($n = 5$ biologically independent replicates) and black asterisks represent significant differences between WT and *atg2*(*p1*) or *atg7*(*p4*) (*$P < 0.01$ two-sided Student's *t*-test). Fifteen leaves were tested. **c**, Detection of ROS in leaf mesophyll cells of WT, *atg2*(*p1*), and *atg7*(*p4*) plants stained with H_2-DCF (green) after exposure to the low- and high-intensity light for 16 h. Note that most peroxisomes (RFP-PTS1,

magenta) in a large aggregate and small parts of the chloroplast (chlorophyll autofluorescence, blue) were stained with H_2-DCF. Scale bars, 10 µm. **d**, The fluorescence intensity of H_2-DCF in peroxisomes of WT, *atg2*(*p1*), and *atg7*(*p4*) plants (**c**). A total of 100 peroxisomes in each line were tested. The error bars indicate mean ± standard deviation (n = 8 biologically independent replicates) and asterisks indicate significant differences between the low- and high-intensity light in WT and *atg2*(*p1*) or *atg7*(*p4*) (*P < 0.01, two-sided Student's *t*-test). **e**, Ratio of peroxisome aggregates stained with H_2-DCF to total peroxisome aggregates. Number of aggregates: WT (n = 11), *atg2*(*p1*) (n = 89), and *atg7*(*p4*) (n = 49). **f**, Relative intensity of H_2-DCF in peroxisomes compared to that in chloroplasts. Number of cells: WT (n = 151), *atg2*(*p1*) (n = 140), and *atg7*(*p4*) (n = 135). The error bars indicate mean ± standard deviation (n = at least 6 biologically independent replicates) and black asterisks represent significant differences between WT and *atg2*(*p1*) or *atg7*(*p4*) (*P < 0.01, **P < 0.05, two-sided Student's *t*-test) in (**e**, **f**). The representative images of H_2-DCF-stained leaf cells in (**c**) show a summary of at least three repeated experiments.

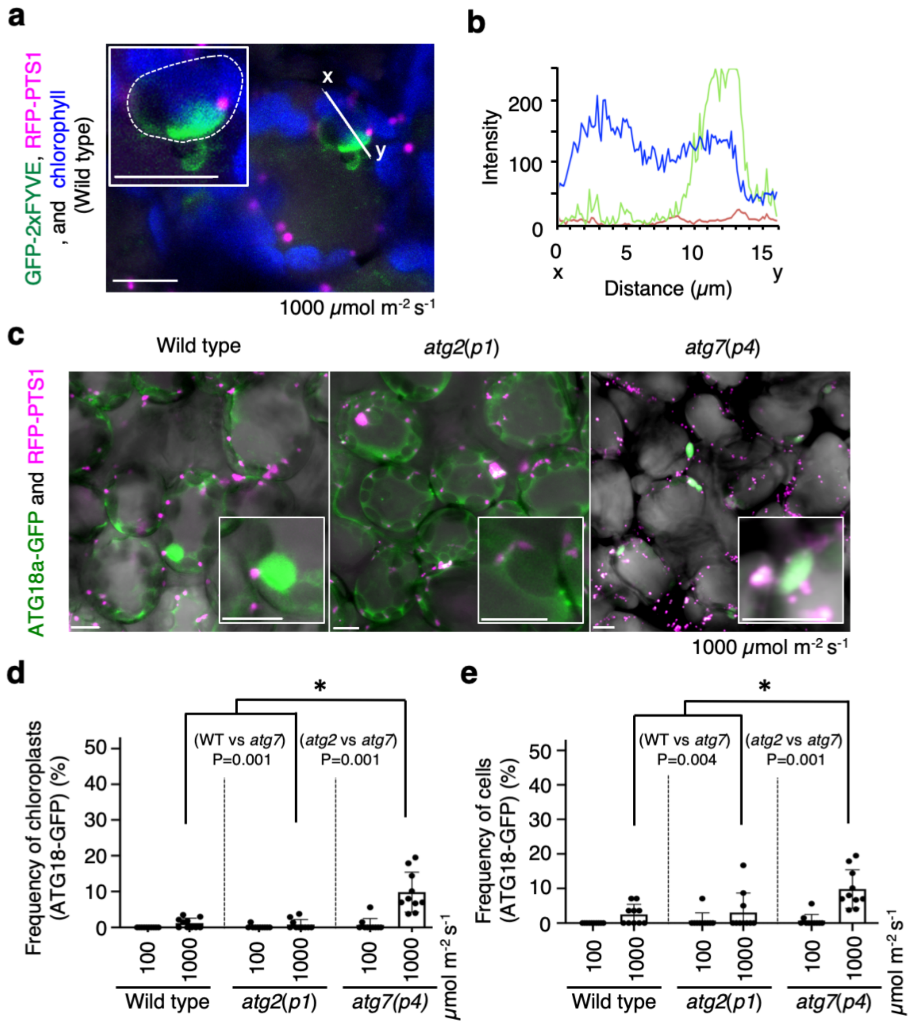

Supplementary Figure 31. Accumulation of ROS in the chloroplasts of *atg2* and *atg7* in high-intensity light

a, Chloroplasts (chlorophyll autofluorescence, blue) targeted by GFP-2×FYVE (green) in a leaf mesophyll cell of a wild-type (WT) plant expressing *RFP-PTS1*(magenta). A chloroplast targeted by GFP-2×FYVE is surrounded by a white-dashed line. The images are obtained under the same condition as described in **Supplementary Fig. 30**. Scale bars, 10 µm. **b**, Plot profile of fluorescence on white line x-y across a chloroplast (blue line) surrounded by GFP-2×FYVE (green line) in the image in (**a**). **c,** Chloroplasts (grey globular structures) targeted by ATG18a-GFP (green) in leaf mesophyll cells of WT, *atg2*(*p1*), and *atg7*(*p4*) plants expressing *RFP-PTS1*(magenta). Scale bars, 5 µm. **d**, Frequency of chloroplasts targeted by ATG18a-GFP to total number of chloroplasts (*$P < 0.01$, two-sided Student's *t*-test). Number of tested chloroplasts: WT, low light (n = 649); WT, high-intensity light (n = 628); *atg2*(*p1*), low light (n = 651); *atg2*(*p1*) high-intensity light (n = 605); *atg7*(*p4*), low light (n = 812); and *atg7*(*p4*) high-intensity light (n = 479). **e,** Cells containing ATG18a-GFP-targeted chloroplasts to total number of cells. Number of tested cells: WT, low light (n = 110); WT, high-intensity light (n = 280); *atg2*(*p1*), low light (n = 190); *atg2*(*p1*), high-intensity light (n = 190); *atg7*(*p4*), low light (n = 234); and *atg7*(*p4*), high-intensity light (n = 298). The error bars indicate mean ± standard deviation (n = 10 biologically independent replicates) and asterisks represent significant differences between wild type and *atg2*(*p1*) or *atg7*(*p4*) (*$P < 0.01$, two-sided Student's *t*-test) in (**d**, **e**). The representative images in (**a**, **c**) show a summary of five repeated experiments.

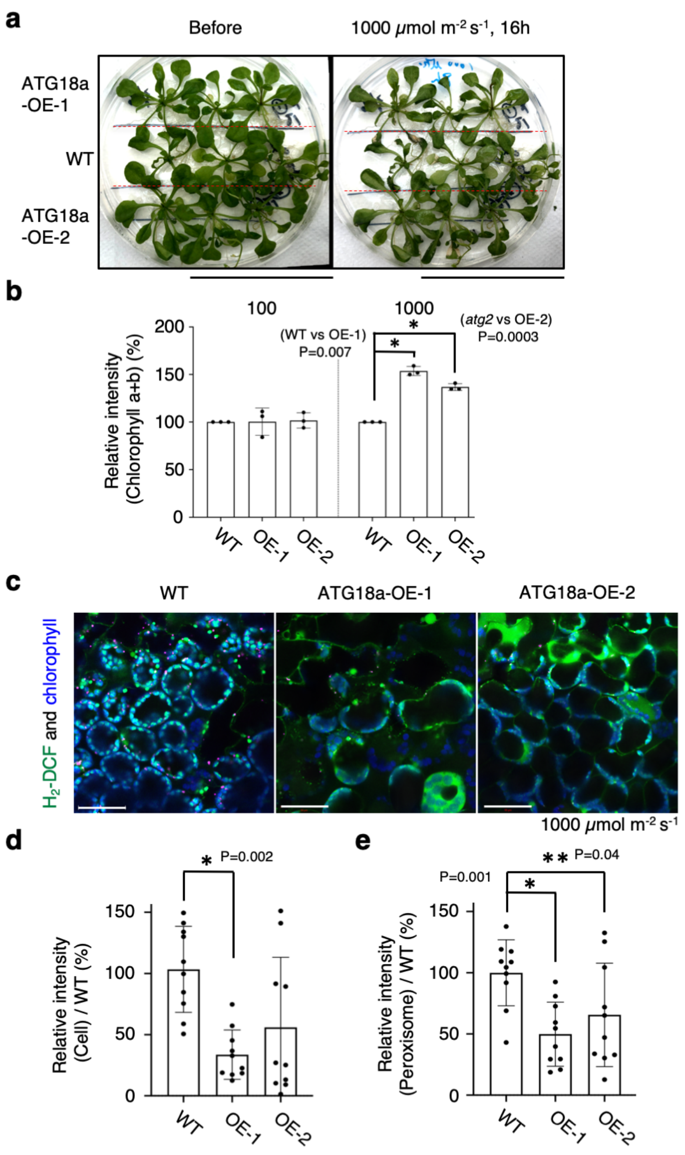

Supplementary Figure 32. Suppression of ROS accumulation in leaf cells of the ATG18a-GFP-overexpressed plants

a, Wild-type (WT: RFP-PTS1) and independent ATG18a-GFP-overexpressed plant lines (ATG18a-OE-1(OE-1), ATG18a-OE-2(OE-2)) before and after adaptation to high-intensity (1000 μmol m^{-2} s^{-1}) light produced using an LED equipment (blue and red light) for 16 h. Three-week-old plants cultured on an agar plate containing ½ MS with 1% sucrose under normal-intensity (100 μmol m^{-2} s^{-1}) white-light conditions were moved to each light condition (*n* = 3 technically independent experiments). Scale bars, 5 cm. **b,** Relative chlorophyll a + b levels in the leaves of WT, OE-1, and OE-2 leaves grown under low (100 μmol m^{-2} s^{-1}) and high (1000 μmol m^{-2} s^{-1}) intensity light conditions. The chart shows mean ± standard deviation of three replications (*n* = 12 biologically independent leaves). **c,** Detection of ROS using H_2-DCF staining in leaf cells exposed to high-intensity light (**a**) (*n* = 3 biologically independent replicates). Scale bars, 50 μm. **d, e,** Relative fluorescence intensity of H_2-DCF in the leaf cells (**c**) and peroxisomes (**d**) of OE-1 and OE-2 compared to that of WT in (**b**). The 15 images captured with CLSM and more than 150 cells were examined from three different experiments. Asterisks indicate significant differences between wild-type and OE-1 or OE-2 (*P < 0.01, **P < 0.05) in two-sided Student's *t*-test (**b**, **d**, **e**).

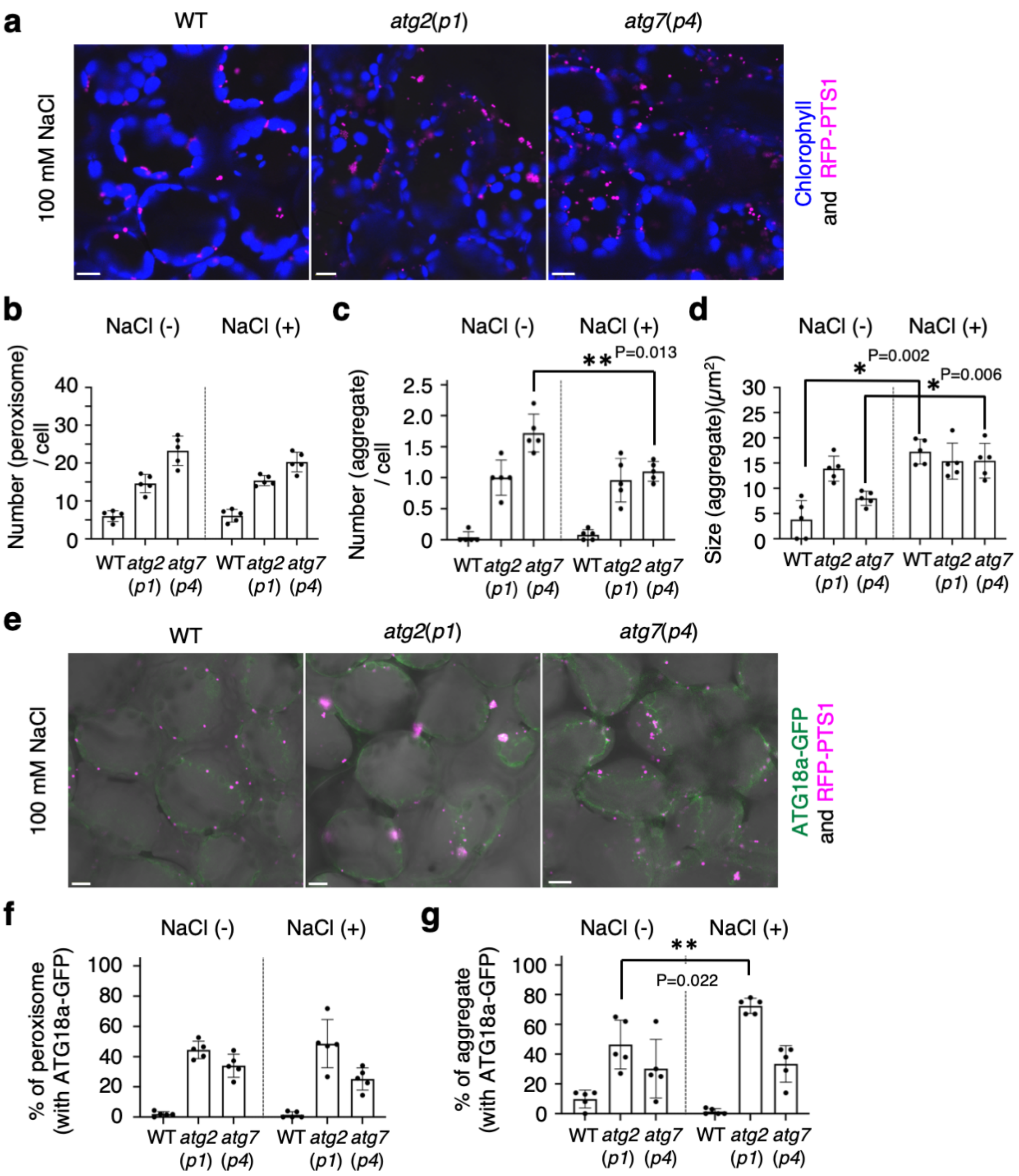

Supplementary Figure 33. Effect of NaCl on pexophagy under normal light

a, CLSM images of peroxisomes (magenta) and chloroplasts (blue) in leaf mesophyll cells from wild-type (WT: RFP-PTS1), *atg2*(*p1*), and *atg7*(*p4*) plants grown on an agar plate containing 100 mM NaCl (NaCl) (n = 5 technically independent replicates). One-week-old plants cultured on an agar plate containing ½ MS with 1% sucrose under normal-intensity (100 μmol m^{-2} s^{-1}) white-light conditions were moved to the agar plate containing 100 mM NaCl and subsequently cultured for 2 weeks under the same light conditions. **b–d,** The number of peroxisomes (**b**), peroxisome aggregates (**c**), and the size of peroxisome aggregates (**d**). **e**, CLSM images of peroxisomes (magenta) and ATG18a-GFP (green) in leaf mesophyll cells from WT (RFP-PTS1), *atg2*(*p1*), and *atg7*(*p4*) plants grown on an agar plate containing 100 mM NaCl (NaCl) (n = 5 biologically independent replicates). **f,** Ratio of peroxisomes bound by ATG18a-GFP to total peroxisomes in (**e**). **g,** Ratio of peroxisome aggregates bound by ATG18a-GFP to total peroxisome aggregates in (**e**). The error bars indicate mean ± standard deviation (n = 5 biologically independent replicates and n = 200 biologically independent cells) and asterisks represent significant differences between wild type and *atg2*(*p1*) or *atg7*(*p4*) with or without NaCl (*$P < 0.01$, **$P < 0.05$, two-sided Student's t-test) in (**b–d**, **f**, **g**). Scale bars in (**a**, **e**), 10 μm.

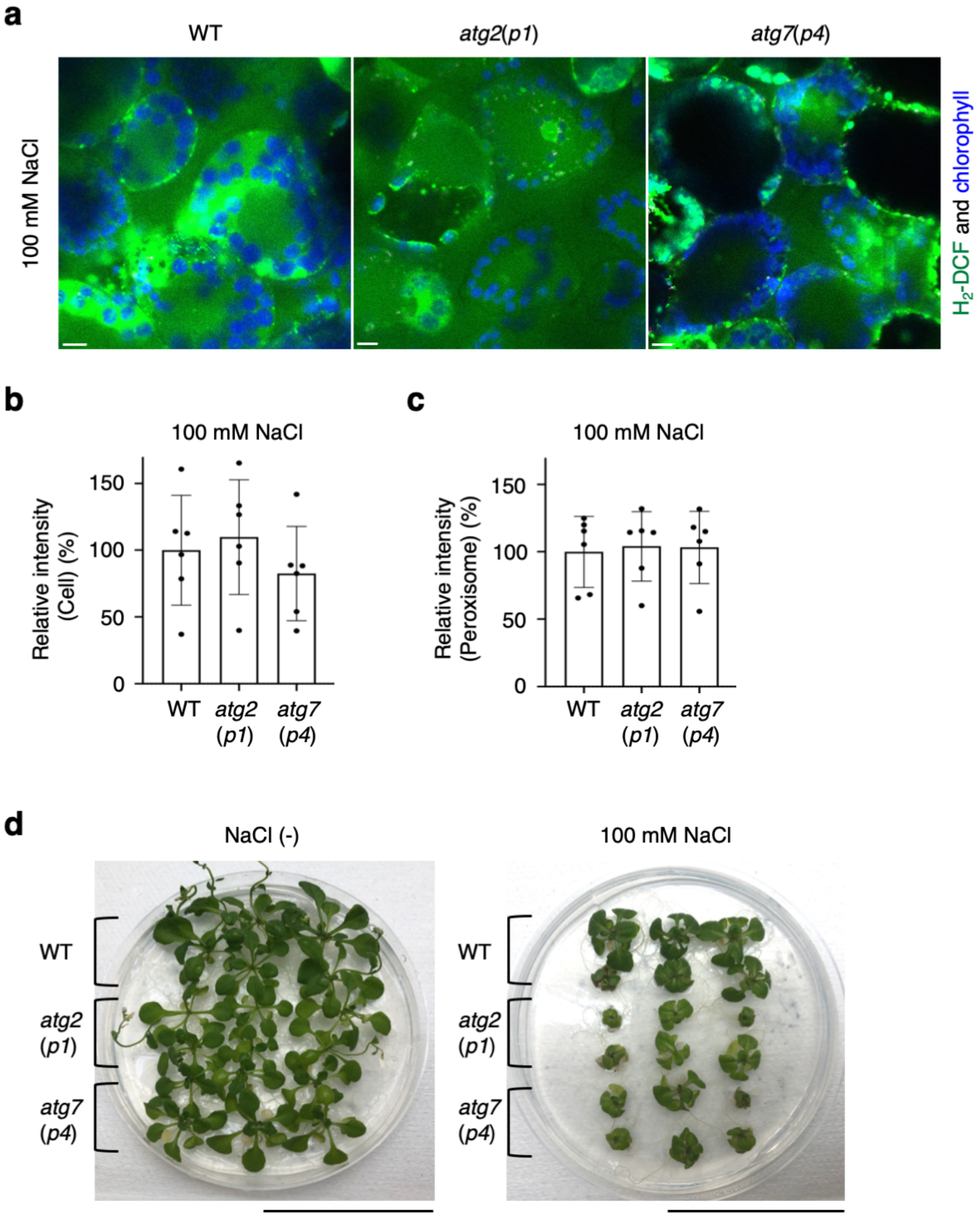

Supplementary Figure 34. ROS accumulation in leaf cells of *atg2* and *atg7* under salt stress

a, Detection of ROS by H_2-DCF staining (green) in mesophyll cells from wild-type (WT), *atg2*(*p1*), and *atg7*(*p4*) leaf cells cultured with 100 mM NaCl under normal intensity (100 µmol m^{-2} s^{-1}) white-light conditions. The representative images show a summary of at least six independent replicates. Scale bars, 10 µm. **b, c**, Relative fluorescence intensity of H_2-DCF in leaf mesophyll cells (**b**) and peroxisomes (**c**) of *atg2*(*p1*) and *atg7*(*p4*) compared to those of WT. The error bars indicate mean ± standard deviation (n = 6 biologically independent replicates and independent 30 cells) in (**b**, **c**). There was no significant difference between WT and *atg2* or *atg7*. **d,** WT, *atg2*(*p1*), and *atg7*(*p4*) plants were grown on an agar plate containing 100 mM NaCl for 2 weeks under the normal white-light condition after cultivation for one week on the NaCl-free agar plate (n = 3 technically independent replicates). Scale bars, 5 cm.

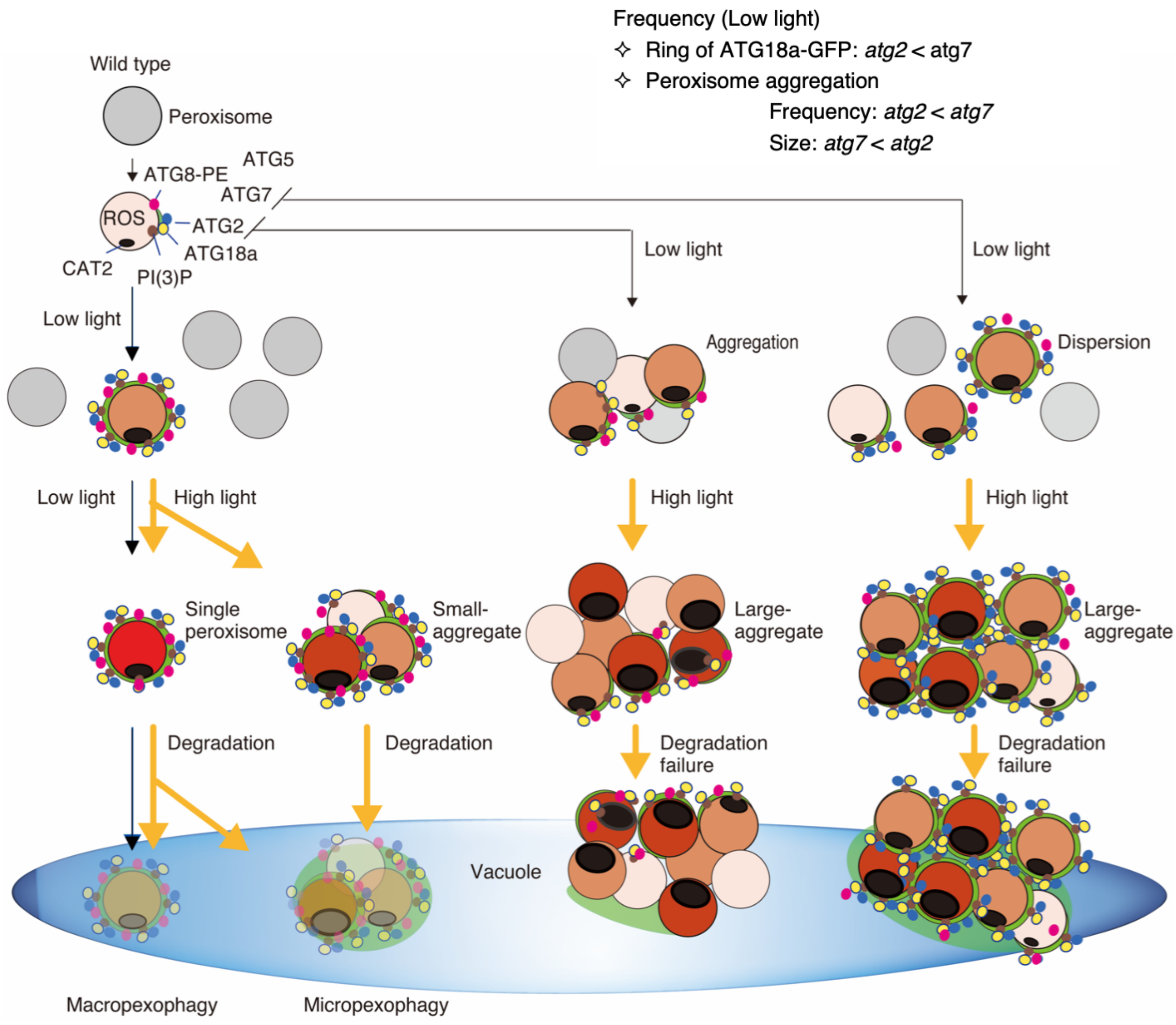

Supplementary Figure 35. Model for processing pexophagosome formation in *atg2* and *atg7* plants

In *atg7* plants, peroxisomes are enveloped by both GFP-2×FYVE and ATG18a-GFP, whereas peroxisomes in *atg2* plants are not. This may generate different phenotypes of peroxisome aggregation between *atg2* and *atg7* plants: peroxisomes in *atg2* form strong aggregates, whereas those in *atg7* disperse and show weak aggregation under low and normal intensity (100 μmol m^{-2} s^{-1}) white light condition (see **Fig. 1** and **Supplementary Fig. 1**). ATG18a and ATG2 are essential for the initiation of pexophagosome formation. ATG7 is involved in the maturation of pexophagosomes by synthesizing the ATG8-PE conjugate on the membrane of pexophagosomes, which are transported to vacuoles for degradation[11,14]. Both *atg2* and *atg7* form a large aggregate of peroxisomes with a high accumulation of catalase and ROS under high-intensity (1000 μmol m^{-2} s^{-1}) light, which is preferentially targeted by GFP-2×FYVE and ATG18a-GFP (see **Figs. 4–7** and **Supplementary Figs. 21**, **22**, **30**). In *atg7* plants, but not in *atg2* plants, the peroxisome aggregate surrounded by ATG18a-GFP and a vacuolar membrane similar to the way in microautophagy (see **Figs. 5f–j**, **6f–i**); however, it cannot be degraded without ATG8-PE[11,14].

Supplementary Table 1.

Number of ATG18a-GFP or GFP-2×FYVE structures on peroxisomes; the structures are categorised into three types: dot, cup, and ring. The data are derived from ten independent images and shown as the density at 100 × 100 μm^2.

ATG18a-GFP 10000 (μm^2)

	Dot	Cup	Ring	Total	Number
Wild type	1.37	1.13	0.56	3.06	10
atg2(*p1*)	14.87	2.65	0.61	18.14	10
atg7(*p4*)	8.73	2.02	4.40	15.15	10

GFP-2×FYVE 10000 (μm^2)

	Dot	Cup	Ring	Total	Number
Wild type	0.43	0.51	2.21	3.15	10
atg2(*p1*)	36.28	6.85	0.05	43.19	10
atg7(*p4*)	27.74	1.08	0.59	29.40	10

Supplementary Table 2.

List of identified proteins by MS analysis for immunoprecipitation with ATG18a-GFP (*atg2*(*p1*))

	prot_acc	prot_desc	significant score (a)	molecular mass (b)	count of spectrum matches (c)	significant spectrum matches (d)
PM	AT3G16530.1	| Symbols: | Legume lectin family protein | chr3:5624586-5625416 REVERSE LENGTH=276	204	30547	10	8
(Cell wall)	AT3G15356.1	| Symbols: | Legume lectin family protein | chr3:5174603-5175418 REVERSE LENGTH=271	79	29788	4	4
	AT3G53420.1	| Symbols: PIP2A, PIP2, PIP2;1 | plasma membrane intrinsic protein 2A | chr3:19803906-19805454 REVERSE LENGTH=287	43	30683	2	2
	AT5G20630.1	| Symbols: GLP3, GLP3A, GLP3B, ATGER3, GER3 | germin 3 | chr5:6975315-6975950 REVERSE LENGTH=211	37	21993	1	1
	AT2G01210.1	| Symbols: | Leucine-rich repeat protein kinase family protein | chr2:119509-121734 REVERSE LENGTH=716	35	79284	23	1
	AT1G01620.1	| Symbols: PIP1C, TMP-B, PIP1;3 | plasma membrane intrinsic protein 1C | chr1:225986-227176 REVERSE LENGTH=286	23	30841	1	1
Cytoplasm	AT5G09810.1	| Symbols: ACT7 | actin 7 | chr5:3052809-3054220 FORWARD LENGTH=377	121	41937	4	4
	AT3G46520.1	| Symbols: ACT12 | actin-12 | chr3:17128567-17129981 FORWARD LENGTH=377	72	41996	4	3
	AT5G14740.1	| Symbols: CA2, CA18, BETA CA2 | carbonic anhydrase 2 | chr5:4758257-4762382 FORWARD LENGTH=331	69	37105	1	1
	AT1G23410.1	| Symbols: | Ribosomal protein S27a / Ubiquitin family protein | chr1:8314940-8315410 FORWARD LENGTH=156	35	17889	2	1
	AT2G02930.1	| Symbols: ATGSTF3, GST16, GSTF3 | glutathione S-transferase F3 | chr2:851348-852106 REVERSE LENGTH=212	34	24106	1	1
	AT3G19050.1	| Symbols: POK2 | phragmoplast orienting kinesin 2 | chr3:6578047-6590106 FORWARD LENGTH=2771	31	317659	36	1
	AT1G10890.1	| Symbols: | unknown protein; FUNCTIONS IN: molecular_function unknown; INVOLVED IN: biological_process unknown; LOCATED IN: chloroplast; EXPRESSED IN: petal, flower, leaf; EXPRESSED DURING: LP.04 four leaves visible, 4 anthesis, petal differentiation and expansion stage; BEST Arabidopsis thaliana protein match is: unknown protein (TAIR:AT5G13340.1); Has 11769 Blast hits to 8435 proteins in 698 species: Archae - 22; Bacteria - 971; Metazoa - 5937; Fungi - 1065; Plants - 592; Viruses - 101; Other Eukaryotes - 3081 (source: NCBI BLink). | chr1:3628081-3630545 FORWARD LENGTH=288	22	34607	2	1
	AT5G57950.1	| Symbols: | 26S proteasome regulatory subunit, putative | chr5:23460765-23462128 FORWARD LENGTH=227	22	24361	17	1
	AT4G29770.1	| Symbols: | Target of trans acting-siR480/255. | chr4:14576371-14577465 FORWARD LENGTH=277	21	31737	8	2
Vacuole	AT3G58810.1	| Symbols: MTPA2, ATMTPA2, MTP3, ATMTP3 | metal tolerance protein A2 | chr3:21750551-21751849 FORWARD LENGTH=432	15	47928	4	2
Unknown	AT1G04090.1	| Symbols: | Plant protein of unknown function (DUF946) | chr1:1057225-1059247 FORWARD LENGTH=572	36	64955	1	1
	AT1G67050.1	| Symbols: | unknown protein; BEST Arabidopsis thaliana protein match is: unknown protein (TAIR:AT5G38320.1); Has 617 Blast hits to 318 proteins in 80 species: Archae - 0; Bacteria - 16; Metazoa - 141; Fungi - 62; Plants - 128; Viruses - 2; Other E	34	28909	9	4
	AT5G50530.1	| Symbols: | CBS / octicosapeptide/Phox/Bemp1 (PB1) domains-containing protein | chr5:20571876-20574922 REVERSE LENGTH=548	32	60060	4	3
	AT1G49780.1	| Symbols: PUB26 | plant U-box 26 | chr1:18429024-18430289 REVERSE LENGTH=421	26	46561	7	1
HSP	AT5G02500.1	| Symbols: HSC70-1, HSP70-1, AT-HSC70-1, HSC70 | heat shock cognate protein 70-1 | chr5:554055-556334 REVERSE LENGTH=651	437	71712	22	19
	AT3G09440.1	| Symbols: | Heat shock protein 70 (Hsp 70) family protein | chr3:2903434-2905632 REVERSE LENGTH=649	329	71559	19	16
	AT5G02490.1	| Symbols: | Heat shock protein 70 (Hsp 70) family protein | chr5:550296-552565 REVERSE LENGTH=653	325	71741	19	16
	AT5G28540.1	| Symbols: BIP1 | heat shock protein 70 (Hsp 70) family protein | chr5:10540665-10543274 REVERSE LENGTH=669	108	73869	10	5
	AT4G24280.1	| Symbols: cpHsc70-1 | chloroplast heat shock protein 70-1 | chr4:12590094-12593437 FORWARD LENGTH=718	52	76575	3	2
Proteosome	AT1G23410.1	| Symbols: | Ribosomal protein S27a / Ubiquitin family protein | chr1:8314940-8315410 FORWARD LENGTH=156	35	17889	2	1
	AT1G49780.1	| Symbols: PUB26 | plant U-box 26 | chr1:18429024-18430289 REVERSE LENGTH=421	26	46561	7	1
	AT5G57950.1	| Symbols: | 26S proteasome regulatory subunit, putative | chr5:23460765-23462128 FORWARD LENGTH=227	22	24361	17	1
Lipid(membrane)	AT3G15730.1	| Symbols: PLDALPHA1, PLD | phospholipase D alpha 1 | chr3:5330835-5333474 FORWARD LENGTH=810	166	92246	15	9
	AT1G07920.1	| Symbols: | GTP binding Elongation factor Tu family protein | chr1:2455559-2457001 FORWARD LENGTH=449	98	49813	5	4
	AT3G06650.1	| Symbols: ACLB-1 | ATP-citrate lyase B-1 | chr3:2079247-2082633 REVERSE LENGTH=608	34	66342	1	1
Autophagy						

	prot_acc	prot_desc	significant score (a)	molecular mass (b)	count of spectrum matches (c)	significant spectrum matches (d)
Nucleus	AT3G49490.1	| Symbols: | unknown protein; Has 722 Blast hits to 186 proteins in 64 species: Archae - 0; Bacteria - 30; Metazoa - 72; Fungi - 48; Plants - 38; Viruses - 0; Other Eukaryotes - 534 (source: NCBI BLink). | chr3:18344926-18348648 REVERSE LENGTH=953	289	106078	10	10
	AT1G63160.1	| Symbols: RFC2 | replication factor C 2 | chr1:23422068-23423771 REVERSE LENGTH=333	64	37066	22	5
	AT4G39680.1	| Symbols: | SAP domain-containing protein | chr4:18414604-18416938 REVERSE LENGTH=633	39	69514	2	2
	AT2G25000.1	| Symbols: WRKY60, ATWRKY60 | WRKY DNA-binding protein 60 | chr2:10629812-10631095 FORWARD LENGTH=271	26	30730	1	1
	AT4G37080.1	| Symbols: | Protein of unknown function, DUF547 | chr4:17474205-17476716 FORWARD LENGTH=597	22	67930	14	2
	AT1G53090.1	| Symbols: SPA4 | SPA1-related 4 | chr1:19783748-19786690 FORWARD LENGTH=794	21	90099	25	1
	AT1G20400.1	| Symbols: | Protein of unknown function (DUF1204) | chr1:7072192-7075838 REVERSE LENGTH=944	21	107183	1	1
	AT5G10710.1	| Symbols: | INVOLVED IN: chromosome segregation, cell division; LOCATED IN: chromosome, centromeric region, nucleus; EXPRESSED IN: 23 plant structures; EXPRESSED DURING: 13 growth stages; CONTAINS InterPro DOMAIN/s: Centromere protein Cenp-O (Inte	20	36454	1	1
Chloroplast	ATCG00490.1	| Symbols: RBCL | ribulose-bisphosphate carboxylases | chrC:54958-56397 FORWARD LENGTH=479	218	53435	9	7
	AT2G39730.1	| Symbols: RCA | rubisco activase | chr2:16570951-16573345 REVERSE LENGTH=474	157	52347	3	3
	AT3G18890.1	| Symbols: | NAD(P)-binding Rossmann-fold superfamily protein | chr3:6511169-6514729 FORWARD LENGTH=641(Tic62)	58	68642	4	2
	AT4G24280.1	| Symbols: cpHsc70-1 | chloroplast heat shock protein 70-1 | chr4:12590094-12593437 FORWARD LENGTH=718	52	76575	3	2
	AT3G50820.1	| Symbols: PSBO2, PSBO-2, OEC33 | photosystem II subunit O-2 | chr3:18891008-18892311 REVERSE LENGTH=331	39	35226	1	1
	AT1G29910.1	| Symbols: CAB3, AB180, LHCB1.2 | chlorophyll A/B binding protein 3 | chr1:10472443-10473246 REVERSE LENGTH=267	37	28266	3	2
	AT4G10340.1	| Symbols: LHCB5 | light harvesting complex of photosystem II 5 | chr4:6408200-6409496 FORWARD LENGTH=280	35	30195	2	2
	AT5G38420.1	| Symbols: | Ribulose bisphosphate carboxylase (small chain) family protein | chr5:15381203-15381978 REVERSE LENGTH=181	25	20622	3	1
	AT1G67090.1	| Symbols: RBCS1A | ribulose bisphosphate carboxylase small chain 1A | chr1:25048465-25049249 REVERSE LENGTH=180	25	20488	2	1
	ATCG00280.1	| Symbols: PSBC | photosystem II reaction center protein C | chrC:33720-35141 FORWARD LENGTH=473	25	52063	1	1
	ATCG00340.1	| Symbols: PSAB | Photosystem I, PsaA/PsaB protein | chrC:37375-39579 REVERSE LENGTH=734	23	82537	1	1
	AT2G45180.1	| Symbols: | Bifunctional inhibitor/lipid-transfer protein/seed storage 2S albumin superfamily protein | chr2:18626377-18626781 FORWARD LENGTH=134	23	14459	1	1
	AT5G58870.1	| Symbols: ftsh9 | FTSH protease 9 | chr5:23770080-23773719 REVERSE LENGTH=806	21	88012	1	1
	AT1G49750.1	| Symbols: | Leucine-rich repeat (LRR) family protein | chr1:18411177-18412779 REVERSE LENGTH=494	21	55171	1	1
	AT5G43390.1	| Symbols: | Uncharacterised conserved protein UCP015417, vWA | chr5:17422940-17424871 REVERSE LENGTH=643	19	73346	1	1
Mitochondria	AT5G58270.1	| Symbols: STA1, ATATM3, ATM3 | ABC transporter of the mitochondrion 3 | chr5:23562168-23567040 FORWARD LENGTH=728	26	80598	1	1
	AT1G19140.1	| Symbols: | FUNCTIONS IN: molecular_function unknown; INVOLVED IN: ubiquinone biosynthetic process; LOCATED IN: mitochondrion; EXPRESSED IN: 24 plant structures; EXPRESSED DURING: 15 growth stages; CONTAINS InterPro DOMAIN/s: COQ9 (InterPro:IPR013	23	34433	1	1
Peroxisome	AT1G20620.1	| Symbols: CAT3, SEN2, ATCAT3 | catalase 3 | chr1:7143142-7146193 FORWARD LENGTH=492	283	57059	15	10
	AT4G35090.1	| Symbols: CAT2 | catalase 2 | chr4:16700937-16703215 REVERSE LENGTH=492	167	57237	12	5
	AT1G20630.1	| Symbols: CAT1 | catalase 1 | chr1:7146812-7149609 FORWARD LENGTH=492	155	57068	8	5
	AT3G14415.1	| Symbols: | Aldolase-type TIM barrel family protein | chr3:4818667-4820748 FORWARD LENGTH=367(GO)	63	40338	6	1
	AT1G68010.1	| Symbols: HPR, ATHPR1 | hydroxypyruvate reductase | chr1:25493418-25495720 FORWARD LENGTH=386	41	42449	1	1
	AT5G09660.1	| Symbols: PMDH2 | peroxisomal NAD-malate dehydrogenase 2 | chr5:2993645-2995551 REVERSE LENGTH=354	35	37688	3	2
	AT2G22780.1	| Symbols: PMDH1 | peroxisomal NAD-malate dehydrogenase 1 | chr2:9689995-9691923 REVERSE LENGTH=354	23	37841	2	1
	AT2G13360.1	| Symbols: AGT, AGT1, SGAT | alanine:glyoxylate aminotransferase | chr2:5539417-5540902 REVERSE LENGTH=401	22	44465	1	1

Supplementary Table 3.
Number of peroxisomes targeted by ATG18a-GFP or GFP-2×FYVE

ATG18a-GFP (n)			
	Peroxisome	Other	Total
Wild type	7	2	9
atg2(*p1*)	84	26	109
atg7(*p4*)	76	21	97

GFP-2×FYVE (n)			
	Peroxisome	Other	Total
Wild type	40	204	244
atg2(*p1*)	133	80	213
atg7(*p4*)	269	307	576

Supplementary Table 4.

Statistical analysis of ATG18a-GFP and GFP-2×FYVE targeting peroxisome aggregates in wild type, *atg2*(*p1*), and *atg7*(*p4*) plants adapted to low (100 μmol m^{-2} s^{-1})- and high (1000 μmol m^{-2} s^{-1})-intensity light conditions. The total number of peroxisome aggregates (**a**), peroxisome aggregates covered with ATG18a-GFP or GFP-2×FYVE (**b**), autophagosome-like structure targeted by ATG18a-GFP (upper) or GFP-2×FYVE (lower) **(c)**, and tested cells (**d**) were counted in wild-type, *atg2*(*p1*), and *atg7*(*p4*) cells expressing *RFP-PTS1* and *ATG18a-GFP* (upper) or *GFP-2×FYVE* (lower) under the low- or high-intensity light conditions; Numbers are the sum of all in repeated experiments ($n \geq 8$). The b'/a', b'/c', and b'/d' represent the average ratio of experimental replications of peroxisome aggregates with micro pexophagosome (**b'**) to the number of peroxisome aggregates (**a'**), (**b'**) to autophagosome-like structure targeted by ATG18a-GFP (upper) or GFP-2×FYVE (lower) (**c'**), and (**b'**) to and tested cells (**d'**), respectively. SD in parenthesis represents the standard deviation. ND, not decidable.

ATG18a-GFP

Light intensity		a	b	c	d	b'/a' (SD)	b'/c' (SD)	b'/d' (SD)
100 μmol m^{-2} s^{-1}	Wild type	1	0	2	121	0.00	0.00	0.00
	atg2(*p1*)	102	1	2	162	0.01 (0.03)	0.10 (0.32)	0.03 (0.03)
	atg7(*p4*)	102	26	44	213	0.24 (0.13)	0.54 (0.22)	0.12 (0.07)
1000 μmol m^{-2} s^{-1}	Wild type	19	8	19	148	0.43 (0.39)	0.46 (0.30)	0.07 (0.07)
	atg2(*p1*)	66	15	18	151	0.23 (0.14)	0.83 (0.24)	0.11 (0.08)
	atg7(*p4*)	97	58	91	154	0.62 (0.17)	0.63 (0.17)	0.43 (0.18)

GFP-2×FYVE

Light intensity		a	b	c	d	b'/a' (SD)	b'/c' (SD)	b'/d' (SD)
100 μmol m^{-2} s^{-1}	Wild type	10	0	1	225	0.00	ND	0.00
	atg2(*p1*)	95	0	0	119	0.00	ND	0.00
	atg7(*p4*)	49	0	0	142	0.00	ND	0.00
1000 μmol m^{-2} s^{-1}	Wild type	9	1	2	155	ND	ND	ND
	atg2(*p1*)	103	2	3	135	0.02 (0.05)	0.67 (0.58)	0.02 (0.04)
	atg7(*p4*)	108	10	17	193	0.12 (0.10)	0.81 (0.75)	0.07 (0.06)

Supplementary Table 5.

Statistical analysis of Venus-VAM3 targeting peroxisomes in wild-type, *atg2*(*p1*), and *atg7*(*p4*) cells under different light conditions. The total number of peroxisomes aggregates (**a**), peroxisome aggregates with micro pexophagosome (**b**), autophagosome-like structure targeted by Venus-VAM3 (**c**), and tested cells (**d**) were counted in wild type, *atg2*(*p1*), and *atg7*(*p4*) cells expressing *Venus-VAM3* and *RFP-PTS1* under low (100 μmol m^{-2} s^{-1}) - and high (1000 μmol m^{-2} s^{-1}) -intensity light conditions; Numbers are the sum of all in repeated experiments (n ≥ 9). The b'/a', b'/c', and b'/d' represent the average ratio of experimental replications of peroxisome aggregates with micro pexophagosome (**b'**) to the number of peroxisome aggregates (**a'**), (**b'**) to autophagosome-like structure targeted by Venus-VAM3 (**c'**), and (**b'**) to tested cells (**d'**), respectively. SD in parenthesis represents the standard deviation. ND, not decidable.

Venus-VAM3 (100 μmol m^{-2} s^{-1})

	a	b	c	d	b'/a' (SD)	b'/c' (SD)	b'/d' (SD)
Wild type	9	3	7	314	0.31 (0.46)	0.50 (0.55)	0.01 (0.02)
atg2(*p1*)	340	11	20	311	0.03 (0.30)	ND	0.03 (0.03)
atg7(*p4*)	86	29	51	289	0.37 (0.13)	0.55 (0.12)	0.10 (0.05)

Venus-VAM3 (1000 μmol m^{-2} s^{-1})

	a	b	c	d	b'/a' (SD)	b'/c' (SD)	b'/d' (SD)
Wild type	18	11	14	318	0.36 (0.45)	0 (0)	0.03 (0.06)
atg2(*p1*)	170	3	3	204	0.02 (0.04)	ND	0.01 (0.02)
atg7(*p4*)	122	66	91	180	0.54 (0.09)	0.71 (0.18)	0.35 (0.12)

Supplementary Table 6.

Statistical analysis of cells containing peroxisomes targeted by ATG18a-GFP under different light conditions. The total number of cells targeted by ATG18a-GFP was counted in wild-type, *atg2*(*p1*), and *atg7*(*p4*) plants expressing *RFP-PTS1* and *ATG18a-GFP* under low (100 µmol m^{-2} s^{-1}) - and high (1000 µmol m^{-2} s^{-1}) - intensity light conditions. SD in parenthesis represents the standard deviation.

Ratio of cell harbouring ATG18a-GFP-targeted peroxisomes

Light intensity	Wild type (SD)	*atg2*(*p1*) (SD)	*atg7*(*p4*) (SD)
100 µmol m^{-2} s^{-1}	0.144 (0.09)	0.64 (0.10)	0.53 (0.07)
1000 µmol m^{-2} s^{-1}	0.761 (0.16)	0.92 (0.11)	0.88 (0.07)
