# Supplementary material for: Pexophagy suppresses ROS-induced damage in leaf cells under high-intensity light
Source: Nat Commun. 2022 Dec 5;13:7493. doi: 10.1038/s41467-022-35138-z (PMC9722907; doi:10.1038/s41467-022-35138-z)
Supplement: Supplementary file 3 — Description of Additional Supplementary Files [file 41467_2022_35138_MOESM3_ESM.pdf]

Description of Additional Supplementary Files:

Supplementary Movie 1. Wild type in light Time-lapse images of peroxisomes (GFP-PTS1, green) and chloroplasts (autofluorescence, magenta) in wild-type cells after adaptation to white light (100 µmol m−2 s−1) were obtained every 5 s for 250 s using CLSM. Images are stacked at 5 frames per second. Scale bar, 10 µm.

Supplementary Movie 2. The atg2 mutant in light Time-lapse images of peroxisomes (GFP-PTS1, green) and chloroplasts (autofluorescence, magenta) in atg2(p1) cells after adaptation to white light (100 µmol m−2 s−1) were obtained every 5 s for 250 s using CLSM. Images are stacked at 5 frames per second. Large peroxisome aggregates are formed continuously. Scale bar, 10 µm.

Supplementary Movie 3. The atg7 mutant in light Time-lapse images of peroxisomes (GFP-PTS1, green) and chloroplasts (autofluorescence, magenta) in atg7(p4) cells after adaptation to white light (100 µmol m−2 s−1) were obtained every 5 s for 250 s using CLSM. Images are stacked at 5 frames per second. Peroxisome aggregates are occasionally formed in the cells. Scale bar, 10 µm.

Supplementary Movie 4. Peroxule formation in atg7 in light Time-lapse images of peroxules (GFP-PTS1, green) and chloroplasts (autofluorescence, magenta) in atg7(p4) cells after adaptation to white light (100 µmol m−2 s−1) were obtained every 5 s for 250 s using CLSM. Images are stacked at 5 frames per second. Scale bar, 10 µm. Peroxules (white arrows) are formed from the peroxisome aggregates in atg7(p4) cells.

Supplementary Movie 5. ATG18a-GFP in wild type Time-lapse images of peroxisomes (RFP-PTS1, magenta) and ATG18a-GFP (green) in a wild-type cell after adaptation to white light (100 µmol m−2 s−1) were obtained every 5 s for 200 s using CLSM. Images are stacked at 5 frames per second. The peroxisome is gradually surrounded by ATG18a-GFP and transported to an undefined structure. Scale bar, 10 µm.

Supplementary Movie 6. ATG18a-GFP in atg2 Time-lapse images of peroxisomes (RFP-PTS1, magenta) and ATG18a-GFP (green) in an atg2(p1) cell after adaptation to white light (100 µmol m−2 s−1) were obtained every 5 s for 200 s using CLSM. Images are stacked at 5 frames per second. The peroxisome aggregate is targeted, but not surrounded, by ATG18a-GFP. Scale bar, 10 µm.

Supplementary Movie 7. ATG18a-GFP in atg7 Time-lapse images of peroxisomes (RFP-PTS1, magenta) and ATG18a-GFP (green) in an atg7(p4) cell after adaptation to white light (100 µmol m−2 s−1) were obtained every 5 s for 200 s using CLSM. Images are stacked to 5 frames per second. The peroxisome aggregate surrounded by ATG18a-GFP moves in a cell. Scale bar, 10 µm.

Supplementary Movie 8. FRAP analysis (ATG18a-GFP in atg2) Time-lapse images of FRAP analysis of peroxisome aggregate (RFP-PTS1, magenta) targeted by ATG18a-GFP (green) in atg2(p1) cells were obtained using CLSM every 1 s for 33 s from before photobleaching to after fluorescence recovery. Photobleaching was performed at the spots indicated by the red circle. Scale bar, 10 µm.

Supplementary Movie 9. FRAP analysis (ATG18a-GFP in atg7) Time-lapse images of FRAP analysis of peroxisome aggregate (RFP-PTS1, magenta) targeted by ATG18a-GFP (green) in atg7(p4) cells were obtained using CLSM every 1 s for 60 s from before photobleaching to after fluorescence recovery. Photobleaching was performed at the spots indicated by the red circle. Scale bar, 10 µm.

Supplementary Movie 10. GFP-2×FYVE in wild type. Time-lapse images of peroxisome (RFP-PTS1, magenta) and GFP-2×FYVE (green) in a wild-type cell after adaptation to white light (100 µmol m^{-2} s^{-1}) were obtained every 5 s for 200 s using CLSM. Images are stacked at 5 frames per second. The peroxisome is gradually surrounded by GFP-2×FYVE. Scale bar, 10 µm.

Supplementary Movie 11. GFP-2×FYVE in atg7 Time-lapse images of peroxisome (RFP-PTS1, magenta) and GFP-2×FYVE (green) in an atg7(p4) cell after adaptation to white light (100 µmol m^{-2} s^{-1}) were obtained every 5 s for 200 s using CLSM. Images are stacked to 5 frames per second. The peroxisome aggregate surrounded by GFP2×FYVE moves in a cell. Scale bar, 5 µm.

Supplementary Movie 12. Z-section images of a leaf mesophyll cell in low-intensity light Z-section images of a leaf mesophyll cell with peroxisomes (RFP-PTS1, magenta) and chloroplast (blue) in wild-type cells under low-intensity (100 µmol m^{-2} s^{-1}) light were obtained using CLSM every 0.5 µm for 12 µm. Peroxisomes are not present in vacuoles. Scale bar, 10 µm.

Supplementary Movie 13. Z-section images of a leaf mesophyll cell treated with concanamycin A in low-intensity light Z-section images of a leaf mesophyll cell with peroxisomes (RFP-PTS1, magenta) and chloroplast (blue) in a wild-type cell treated with concanamycin A under low-intensity (100 µmol m^{-2} s^{-1}) light were obtained using CLSM every 0.5 µm for 12 µm. A few peroxisomes are present in a vacuole. Scale bar, 10 µm.

Supplementary Movie 14. Z-section images of a leaf mesophyll cell in high-intensity light Z-section images of a leaf mesophyll cell with peroxisomes (RFP-PTS1, magenta) and chloroplast (blue) in a wild-type cell under high-intensity (1000 µmol m^{-2} s^{-1}) light were obtained using CLSM every 0.5 µm for 12 µm. A few peroxisomes are in the vacuole. Scale bar, 10 µm.

Supplementary Movie 15. Z-section images of a leaf mesophyll cell treated with concanamycin A in high-intensity light Z-section images of a leaf mesophyll cell with peroxisomes (RFP-PTS1,

magenta) and chloroplast (blue) in a wild-type cell treated with concanamycin A under high-intensity (1000 μmol m−2 s−1) light were obtained using CLSM every 0.5 μm for 12 μm. A few peroxisomes are in the vacuole. Scale bar, 10 μm.

Supplementary Movie 16. The vacuolar cavity in wild type Time-lapse images of peroxisomes (RFP-PTS1, magenta) and vacuolar membranes (VenusVAM3, green) from a wild-type cell after adaptation to white light (100 μmol m−2 s−1) were obtained using CLSM every 5 s for 250 s. Peroxisomes are not surrounded by vacuolar structures (white arrows) similar to bulbs. Scale bar, 10 μm.

Supplementary Movie 17. The vacuolar cavity in atg7 Time-lapse images of peroxisomes (RFP-PTS1, magenta) and vacuolar membranes (VenusVAM3, green) in atg7(p4) cells after adaptation to white light (100 μmol m−2 s−1) were obtained using CLSM every 5 s for 250 s. Peroxisomes are surrounded by vacuolar structures (white arrows) similar to bulbs. Scale bar, 5 μm.

Supplementary Movie 18. Peroxisome on isolated vacuole of wild type expressing VenusVAM3. Time-lapse images of peroxisomes (RFP-PTS1, magenta) and Venus-VAM3 (green) on isolated vacuoles from a wild-type cell after adaptation to white light (100 μmol m−2 s−1) were obtained every 5 s for 150 s using CLSM. Images are stacked at 1 frame per second. The peroxisomes surrounded by Venus-VAM3 were transported inside the vacuole. Scale bar, 10 μm.

Supplementary Movie 19. Peroxisome on isolated vacuole of atg7(p4) expressing VenusVAM3 Time-lapse images of peroxisomes (RFP-PTS1, magenta) and Venus-VAM3 (green) on isolated vacuoles from an atg7(p4) cell after adaptation to high-intensity (1000 μmol m−2 s−1) light were obtained every 5 s for 150 s using CLSM. Images are stacked at 1 frame per second. The peroxisomes and peroxisome aggregate surrounded by Venus-VAM3 were not transported inside the vacuole. Scale bar, 10 μm.

Supplementary Movie 20. Nomarski image of the peroxisome on isolated vacuole of atg7(p4) expressing Venus-VAM3 Time-lapse images of peroxisomes on the surface of the isolated vacuole from an atg7(p4) cell after adaptation to high-intensity (1000 μmol m−2 s−1) light were obtained every 5 s for 150 s using CLSM. Images are stacked at 1 frame per second. The peroxisomes and peroxisome aggregate were not transported inside the vacuole. Scale bar, 10 μm.

Supplementary Movie 21. Peroxisome on isolated vacuole of wild type expressing ATG18aGFP Time-lapse images of peroxisomes (RFP-PTS1, magenta) and ATG18a-GFP (green) on an isolated vacuole from a wild-type cell after adaptation to high-intensity (1000 μmol m−2 s−1) light were obtained every 5 s for 150 s using CLSM. Images are stacked at 1 frame per second. The peroxisome was not seen on the vacuole. Scale bar, 10 μm.

Supplementary Movie 22. Peroxisome on isolated vacuole of atg7(p4) expressing ATG18aGFP Time-lapse images of peroxisomes (RFP-PTS1, magenta) and ATG18a-GFP (green) on an isolated vacuole from an atg7(p4) cell after adaptation to high-intensity (1000 μmol m^{-2} s^{-1}) light were obtained every 5 s for 150 s using CLSM. Images are stacked at 1 frame per second. The peroxisomes and peroxisome aggregate surrounded by ATG18a-GFP bound on the vacuole surface and not transported inside the vacuole. Scale bar, 10 μm.
