# Supplementary material for: Pexophagy suppresses ROS-induced damage in leaf cells under high-intensity light
Source: Nat Commun. 2022 Dec 5;13:7493. doi: 10.1038/s41467-022-35138-z (PMC9722907; doi:10.1038/s41467-022-35138-z)
Supplement: Supplementary file 26 — Reporting Summary [file 41467_2022_35138_MOESM26_ESM.pdf]

nature portfolio

Corresponding author(s): Kenji Yamada, Mikio Nishimura

Last updated by author(s): Nov 11, 2022

Reporting Summary

Nature Portfolio wishes to improve the reproducibility of the work that we publish. This form provides structure for consistency and transparency in reporting. For further information on Nature Portfolio policies, see our Editorial Policies and the Editorial Policy Checklist.

Statistics

For all statistical analyses, confirm that the following items are present in the figure legend, table legend, main text, or Methods section.

n/a	Confirmed	
☐	☒	The exact sample size (n) for each experimental group/condition, given as a discrete number and unit of measurement
☐	☒	A statement on whether measurements were taken from distinct samples or whether the same sample was measured repeatedly
☐	☒	The statistical test(s) used AND whether they are one- or two-sided<br>*Only common tests should be described solely by name; describe more complex techniques in the Methods section.*
☒	☐	A description of all covariates tested
☐	☒	A description of any assumptions or corrections, such as tests of normality and adjustment for multiple comparisons
☐	☒	A full description of the statistical parameters including central tendency (e.g. means) or other basic estimates (e.g. regression coefficient) AND variation (e.g. standard deviation) or associated estimates of uncertainty (e.g. confidence intervals)
☐	☒	For null hypothesis testing, the test statistic (e.g. F, t, r) with confidence intervals, effect sizes, degrees of freedom and P value noted<br>*Give P values as exact values whenever suitable.*
☒	☐	For Bayesian analysis, information on the choice of priors and Markov chain Monte Carlo settings
☒	☐	For hierarchical and complex designs, identification of the appropriate level for tests and full reporting of outcomes
☐	☒	Estimates of effect sizes (e.g. Cohen's d, Pearson's r), indicating how they were calculated

Our web collection on statistics for biologists contains articles on many of the points above.

Software and code

Policy information about availability of computer code

Data collection	We used Fiji for dara collection. Fiji is an open-source platform for biological-image analysis (Nature methods 9(7): 676-682) and Zen software to collect images on the microscope.
Data analysis	Data analysis were performed using Excel (Microsoft(R) Excel for Mac Ver. 16.16.18 (200112) . Dot plot analysis were performed using Prisms 9 for macOS (version 9.20(283) GraphPad Software, https://www.graphpad.com).

For manuscripts utilizing custom algorithms or software that are central to the research but not yet described in published literature, software must be made available to editors and reviewers. We strongly encourage code deposition in a community repository (e.g. GitHub). See the Nature Portfolio guidelines for submitting code & software for further information.

Data

Policy information about availability of data

All manuscripts must include a data availability statement. This statement should provide the following information, where applicable:

- Accession codes, unique identifiers, or web links for publicly available datasets
- A description of any restrictions on data availability
- For clinical datasets or third party data, please ensure that the statement adheres to our policy

We have raw data sets for all figures and can provide for whenever requirement.

Field-specific reporting

Please select the one below that is the best fit for your research. If you are not sure, read the appropriate sections before making your selection.

- [x] Life sciences
- [ ] Behavioural & social sciences
- [ ] Ecological, evolutionary & environmental sciences

For a reference copy of the document with all sections, see nature.com/documents/nr-reporting-summary-flat.pdf

Life sciences study design

All studies must disclose on these points even when the disclosure is negative.

Sample size

Figures:
Figure 1. (b) We counted peroxisomes in 127 cells in WT, 101 cells in atg2(p1), and 94 cells in atg7(p4). (c,d) At least five independent experiments were performed. More than 100 cells were examined.
Figure 2,3 (b-d). More than 100 cells from five biological replicates were examined.(f) n= 10 biologically independent replicates
Figure 4. (c,d) More than 165 cells were tested (n= 3 technically independent replicates). (f-h) Five different plants were examined.
Figure 5.(c) More than 135 cell were examined. (d) Number of structures examined: WT (n = 8), atg2(p1) (n = 15), and atg7(p4) (n =58) for ATG18a-GFP; WT (n = 1), atg2(p1) (n = 2), and atg7(p4) (n = 10) for GFP-2×FYVE. Figure 5h. More than 200 cells were tested. (i) Size of the vacuolar-membrane structures surrounding the large aggregate of peroxisomes. The numbers of the structures were 11 (WT, high-intensity light) and 66 (atg7(p4), high-intensity light). (c, d, h–j;) At least 8 biologically independent replicates.
Figure 6. (g,h) Twelve vacuoles were examined for each line. (i) The number of structures were counted from each eight vacuole.
Figure 7. (b) Total of 140 peroxisomes in each line were tested (n= 8 biologically independent replicates).

Supplementary Figures :
Supplementary Figure 1. More than 200 cells were tested from ten biologically independent replicates.
Supplementary Figure 2. (a) Growth test of plant (n= 4 technically independent replicates). (b,c) Four independent experiments were performed (n= 5 biologically independent replicates). (e) Relative quantification of NBT intensity were examined using 15 leaves (n= 5 biologically independent replicates), in each condition.
Supplementary Figure 3. (b) Number of tested peroxisomes: wild type (WT) (n = 36), atg2(p1) (n = 134), and atg7(p4) (n = 183). Five different sections were used. (e) Number of tested peroxisomes: GFP (n = 32), MS (n = 22), ICL (n = 15), GO (n = 37), HPR (n = 35), CAT (n = 30). (f) Number of tested DG: GFP-PTS1 (n = 7), atg2(p1) (n = 14), and atg7(p4) (n = 23) (n= 5 technically independent sections).
Supplementary Figure 4. n=3 technically independent experiments
Supplementary Figure 5.The representative images in LSM show a summary of at least eight independent experiments. The representative images in EM show a summary of at least five independent experiments.
Supplementary Figure 6. Seven protoplasts were examined at least 3 independent experiments.
Supplementary Figure 7. Seven protoplasts were examined at least 3 independent experiments.
Supplementary Figure 8. The representative images in (a, b) show a summary of at least three independent experiments.
Supplementary Figure 9. The data are taken from ten sections and shown as the density at 100 × 100 µm2 from at least eight independent experiments.
Supplementary Figure 10. he representative images in (a–c) show a summary of at least ten independent experiments.
Supplementary Figure 11. Five individuals following FRAP analyses were tested.
Supplementary Figure 12. Five individuals following FRAP analyses were tested.
Supplementary Figure 13. The representative images show a summary of at least three independent experiments and two proteome analyses were performed.
Supplementary Figure 14. The representative images in (a–c) show a summary of at least ten independent experiments. n= 3 technically independent replicates).
Supplementary Figure 15-17. The representative images show a summary of at least five
Supplementary Figure 18. The representative images in (a, b) show a summary of at least five repeated experiments. (c) n= 3 technically independent replicates. (d) The representative images are selected from at least five independent experiments.
Supplementary Figure 19. Total 100 peroxisomes from at least nine independent experiments.
Supplementary Figure 20. The representative images in (a) show a summary of at least three independent experiments.
Twelve biologically independent plants from three technically independent replicates were examined.
Supplementary Figures 21(b) and 22 (a,b). More than 100 cells were tested. Each tested number for normal light: WT top (n = 6), WT bottom (n = 28), atg2 top (n = 50), atg2 bottom (n = 63), atg5 top (n = 43), atg5 bottom (n = 65), atg7 top (n = 83), atg7 bottom (n = 90), atg9 top (n = 22), atg9 bottom (n = 18); for high-intensity light in (b): WT top (n = 19), WT bottom (n = 48), atg2 top (n = 52), atg2 bottom (n = 55), atg5 top (n = 45), atg5 bottom (n = 76), atg7 top (n = 42), atg7 bottom (n = 49), atg9 top (n = 40), atg9 bottom (n = 41).
Supplementary Figures 22 (c-e). At least three repeated experiments.
Supplementary Figure 23. Ten cells from eight independent leaves were examined.
Supplementary Figure 24. Ten biological independent replicates were examined.
Supplementary Figure 25.(a,b) The representative images show a summary of five independent experiments. (c) The data are collected from more than 100 cells in CLSM images from at least five independent experiments. (e-i) At least three independent experiments are examined.
Supplementary Figure 26. (b,c) Ten images were tested five repeated experiments.
Supplementary Figures 27 (e), 28 (g,h), and 29 (d). At least nine vacuoles from three repeated experiments were examined.
Supplementary Figure 30. (b) Fifteen leaves form five biological replicates were tested. (d) A total of 100 peroxisomes in each line were tested (five biological replicates). (e) Number of aggregates: WT (n = 11), atg2(p1) (n = 89), and atg7(p4) (n = 49). (f) Five biological replicates were tested.
Supplementary Figure 31.(d,e) Number of tested chloroplasts: WT, low light (n = 649); WT, high-intensity light (n = 628); atg2(p1), low light (n

= 651); atg2(p1) high-intensity light (n = 605); atg7(p4), low light (n = 812); and atg7(p4) high-intensity light (n = 479). e, Number of tested cells: WT, low light (n = 110); WT, high-intensity light (n = 280); atg2(p1), low light (n = 190); atg2(p1), high-intensity light (n = 190); atg7(p4), low light (n = 234); and atg7(p4), high-intensity light (n = 298). Five biological replicates was examined.
Supplementary Figure 32. (b) Three replications where more than 12 leaves were used in one extraction. (d,e) The 15 images captured with CLSM and more than 150 cells were examined from three different experiments.
Supplementary Figure 33. (b-d, f,g) More than 200 cells were tested. Total 100 peroxisomes in ten leaves were examined from at least three experiments.
Supplementary Figure 34. (b,c) The six images captured with CLSM and 30 cells were examined.

Data exclusions	No data were excluded in this study.
Replication	We repeatedly performed experiments more than three times and used distinct samples to verify the repriduciblity of the results in this study.
Randomization	We randamly selected samples in this study for avoiding arbitrary interference.
Blinding	Studies were performed by blinding during data collection with more than two distinct reserchers.

Reporting for specific materials, systems and methods

We require information from authors about some types of materials, experimental systems and methods used in many studies. Here, indicate whether each material, system or method listed is relevant to your study. If you are not sure if a list item applies to your research, read the appropriate section before selecting a response.

Materials & experimental systems

n/a	Involved in the study	
☐	☒	Antibodies
☒	☐	Eukaryotic cell lines
☒	☐	Palaeontology and archaeology
☒	☐	Animals and other organisms
☒	☐	Human research participants
☒	☐	Clinical data
☒	☐	Dual use research of concern

Methods

n/a	Involved in the study	
☒	☐	ChIP-seq
☒	☐	Flow cytometry
☒	☐	MRI-based neuroimaging

Antibodies

Antibodies used	Antibodies against peroxisomal proteins catalase(CAT) , peroxin 14 (PEX14), malate synthase (MS), isocitrate lyase (ICL), glycolate oxidase (GO), hydroxypyruvate reductase (HPR), ascorbate peroxidase (APX) are produced in our laboratory in previous works. We purchased the antibodies against mitochondrial proteins cytochrome c oxidase 2 (COXII) (Agrisera, Sweden) and serine hydroxymethyltransferase (SHMT) (Agrisera, Sweden) from company.
Validation	*Describe the validation of each primary antibody for the species and application, noting any validation statements on the manufacturer's website, relevant citations, antibody profiles in online databases, or data provided in the manuscript.*
